# Runs of homozygosity reveal population dynamics and selection across global cattle

**DOI:** 10.1186/s40104-026-01403-0

**Published:** 2026-05-06

**Authors:** Jun Ma, Xinxia Liu, Xue Gao, Dawei Wei, Zijing Zhang, Abdullah Muhammad, Ningbo Chen, Xiaoting Xia, Yun Ma, Eryao Wang, Xian Liu, Chuzhao Lei, Yu Jiang, Yongzhen Huang

**Affiliations:** 1 College of Animal Science and Technology, Northwest A&F University, Yangling, Shaanxi 712100 People’s Republic of China; 2 Institute of Animal Science, Chinese Academy of Agricultural Sciences, Beijing, 100091 People’s Republic of China; 3 College of Animal Science and Technology, Ningxia University, Yinchuan, Ningxia 750021 People’s Republic of China; 4 Institute of Animal Husbandry, Henan Academy of Agricultural Sciences, Zhengzhou, Henan 450002 People’s Republic of China; 5 Henan Provincial Livestock Technology Promotion Station, Zhengzhou, Henan 450008 People’s Republic of China

**Keywords:** Cattle, Inbreeding coefficients, Population dynamics, Runs of homozygosity, ROH hotspots

## Abstract

**Background:**

Cattle have undergone complex evolutionary trajectories shaped by domestication, migration, and selection. Although runs of homozygosity (ROH) are a ubiquitous genomic feature, their full potential to decipher the evolutionary history and functional consequences in global cattle populations remains underexplored. We analyzed whole-genome sequences from 102 breeds across 17 geographic regions to conduct a global investigation of ROH landscapes, population structure, genomic inbreeding, and functional variants.

**Results:**

ROH patterns revealed elevated homozygosity burdens in intensively selected European breeds, whereas South Chinese indicine showed a high short ROH burden, suggestive of a unique ancient demography. ROH-based principal component analysis (PCA) and admixture delineated taurine-indicine lineages, region-specific ancestries, inbreeding, and breeding effects. ROH-based inbreeding coefficient (*F*_ROH_) exhibited greater stability for cross-population inbreeding assessment, showing a high correlation with excess of homozygosity-based inbreeding coefficient (*F*_HOM_) and a negative association with heterozygosity. Region-specific ROH hotspots, identified via permutation test, reflected a combination of local adaptation and demographic legacies. Trait-focused analyses, cross-validated with multiple selection scans, identified genes underlying growth, milk, and climate adaptation. Notably, we found missense mutations in *CHEK2*, *SPG7*, *FANCA*, and *MSRB3*, whose frequencies were significantly correlated with temperature and humidity.

**Conclusion:**

This study establishes ROH as a pivotal genomic marker for illuminating the dynamics of domestication, migration, inbreeding, and selection. Our findings offer valuable resources and insights for advancing genetic conservation and precision breeding in cattle under the pressures of climate change.

**Supplementary Information:**

The online version contains supplementary material available at 10.1186/s40104-026-01403-0.

## Background

Cattle encompass taurine (*Bos taurus*) and indicine (*Bos indicus*) lineages, originating from the domestication of the aurochs (*Bos primigenius*) approximately 10,000 and 8,000 years ago in the Fertile Crescent and Indus Valley, respectively [1–3]. Human migration, trade, and selective breeding have facilitated the worldwide dissemination of cattle, resulting in a vast array of breeds adapted to diverse environments. Indicine cattle, primarily distributed across South Asia, East Asia, and Africa, have evolved distinct advantages for survival in semi-arid or humid-hot climates [4]. In contrast, European taurine cattle underwent intensive selective breeding following the agricultural revolution, producing commercial breeds for high-output meat and milk [2]. This evolutionary trajectory, driven by both natural and artificial selection, has left distinct genomic signatures of selection and demography.

ROH, defined as contiguous homozygous segments arising from shared ancestry [5], provide valuable insights into the demographic history, inbreeding levels, and selection pressures within populations [6, 7]. Long ROH signals recent consanguinity, whereas short ROH reflects ancient bottlenecks or linkage disequilibrium [8]. Moreover, selection is expected to reduce heterozygosity in trait-critical regions, thereby forming non-random shared ROH hotspots in similar populations that are indicative of selective sweeps rather than mere inbreeding [6], which offer a window into the genetic basis of key economic and adaptive traits in livestock and poultry. In cattle genomics, however, the potential of ROH remains underexplored. Previous studies have predominantly relied on limited-density SNP arrays, which lack the resolution to accurately detect short ROH and rare variants [7, 9–13], limiting insights into the combined effects of ancient demographic events and selection. While whole-genome sequencing (WGS) overcomes these limitations and enables precise ROH detection, existing applications have been largely confined to regional or breed-specific analyses [14–17]. Furthermore, most studies employ fixed empirical frequency thresholds to define ROH hotspots [9, 11, 18, 19], overlooking population-specific demographic backgrounds and risking excessive false positives in small or highly inbred groups. Therefore, a comprehensive global study that integrates statistical robustness with evolutionary interpretability is essential.

We hypothesize that the accumulation of ROH represents temporally stratified genomic markers that reflect geography- and lineage-specific evolutionary trajectories, enabling the dissection of the histories underlying domestication, migration, and selection. Here, we leverage WGS data from 102 cattle breeds spanning 17 geographic regions to conduct a global investigation of ROH. We first systematically characterized the global ROH landscape, evaluated ROH-based population structure, and estimated genomic inbreeding. To ensure statistical rigor amid demographic heterogeneity and unequal sample sizes, we implemented a permutation framework to identify ROH hotspots. These hotspots were subsequently cross-validated using five independent selection signatures to distinguish selection-driven homozygosity and identify candidate variants associated with growth, dairy, and environmental resilience. Our findings reveal how ROH reflect population history, inbreeding, and selection pressures, thereby shaping genetic diversity and trait differentiation, and provide critical insights for conservation and sustainable breeding strategies in diverse agroecological contexts.

## Materials and methods

### Sample collection

Blood samples were collected from 39 Qinchuan and 5 Nanyang cattle in North-Central China for whole-genome sequencing. To maximize the global geographical coverage of the taurine and indicine lineages, we retrieved whole-genome sequencing data of cattle from public databases, ensuring a minimum of four individuals per breed. Furthermore, to mitigate the impact of unequal sample sizes among breeds, we randomly subsampled 50 individuals from breeds with more than 50 samples, resulting in a dataset of 1,479 individuals from 102 breeds (Table S1). Based on geographic origins, breeds were categorized into 17 regional groups, including Western Europe (*n* = 5), Central-South Europe (*n* = 14), Northern Europe (*n* = 2), Southern Europe (*n* = 2), Middle East (*n* = 1), Northwest China (*n* = 3), North Asia (*n* = 1), Xizang, China (*n* = 2), Northeast Asia (*n* = 3), North-Central China (*n* = 8), South-Central China (*n* = 3), Southwest China (*n* = 9), South China (*n* = 9), Southeast Asia (*n* = 2), South Asia (*n* = 13), West Africa (*n* = 2), and East Africa (*n* = 23). Detailed sample information was provided in Table S1.

### SNP quality control and ROH detection

Raw WGS reads were quality-assessed using FastQC v.0.11.8. Adapters and low-quality sequences were removed with Trimmomatic v.0.39 [20]. The high-quality reads were aligned to the bovine reference genome ARS-UCD1.2 (GCF_002263795.1) with the Burrows-Wheeler Aligner (BWA) v.0.7.8 with default parameters [21]. Alignments were converted to BAM format and sorted using SAMtools v.1.3.1 [22]. Potential PCR duplicates were marked and removed with Picard v.2.20.2 (http://broadinstitute.github.io/picard). Sequencing depth and coverage were evaluated using QualiMap v2.2.1 [23].

Single-nucleotide polymorphisms (SNPs) were identified using the Genome Analysis Toolkit (GATK) v4.3.0.0 [24]. Individual Genomic Variant Call Format files were generated with the ‘HaplotypeCaller’ module using the ‘-ERC GVCF’ option, merged with ‘CombineGVCFs’. We proceeded to call and select candidate SNPs from the combined GVCF files using ‘GenotypeGVCFs’ and ‘SelectVariants’, respectively. To minimize false positives, SNPs were filtered using the ‘VariantFiltration’ module following GATK best practices: Quality by Depth (QD) < 2.0, Quality Score (QUAL) < 30.0, Strand Odds Ratio (SOR) > 3.0, Fisher Strand (FS) > 60.0, Mapping Quality (MQ) < 40.0, MQRankSum < −12.5, ReadPosRankSum < −8.0, with a cluster size of 3, and a cluster window size of 10. Non-biallelic SNPs were excluded using BCFtools v1.8 [25], and only autosomal SNPs were retained. Further quality control on the merged SNP dataset was performed with PLINK (v1.90) using the following criteria: individual call rate > 95%, SNP call rate > 95%, minor allele frequency (MAF) > 0.01, and Hardy–Weinberg equilibrium *P*-value > 10e-6. To minimize close relationships, 214 individuals with PI-HAT values ≥ 0.25 were removed [13, 26]. Missing genotypes were imputed using BEAGLE v5.4 with default parameters [27], resulting in 24,488,825 SNPs from 1,265 individuals across 102 breeds for downstream analysis.

### ROH detection

ROH were detected using PLINK v1.9 with a sliding-window approach across autosomes [28]. Parameter settings were informed by previous bovine WGS ROH studies [15, 29–31], and further refined through a one-factor-at-a-time sensitivity analysis across seven representative breeds (Angus, Mishima, Leiqiong, Butana, Qinchuan, Muturu, and Sahiwal) with diverse demographic histories to ensure robustness against varying genetic backgrounds [32–34]. We tested the impact of varying the minimum SNP count (10–150), minimum ROH length (100–1000 kb), allowed heterozygous SNPs (0–5), allowed missing calls (0–5), and SNP density (10–200 kb/SNP) on the resulting key metrics, including total ROH count, average ROH size, average ROH per animal, and average *F*_ROH_of different length classes.

The final detection parameters were set as follows: minimum of 50 consecutive SNPs per ROH, maximum inter-SNP distance < 1 Mb, allowance of up to 5 missing calls and 3 heterozygous SNPs per ROH, and a minimum length threshold of 500 kb to exclude short segments arising from linkage disequilibrium (LD) blocks [13, 35]. To establish uniform ROH length categories (short, medium, long) for cross-breed comparisons, a three-component Gaussian mixture model was fitted to the genome-wide ROH length distribution using the mclust package (v6.1.1) in R v4.1.2 [8, 36].

### ROH distribution and population structure

The cumulative number and cumulative length of ROH were quantified for each breed and geographic group to assess genomic distribution patterns. To evaluate the statistical significance of variations in ROH burden among geographic regions and breeds, we performed Wilcoxon rank-sum tests for independent comparisons between major lineages and geographic groups, such as Western Europe versus other regions, South China indicine versus South Asia indicine, and Eastern Finncattle versus other breeds. To assess whether introgression from wild bovine species contributed to the unique ROH patterns observed in South China [1, 34], we quantified the frequencies of ROHs overlapping with introgression segments and compared them across geographic groups using paired Wilcoxon signed-rank tests. All *P*-values were adjusted using the Benjamini–Hochberg method.

For population structure analysis, ROH segments were recoded as binary presence/absence (1/0) matrices using a custom script. PCA and ancestry estimation were performed using smartPCA v16000 and ADMIXTURE v1.3.0, respectively [37, 38]. These analyses were conducted separately for short, medium, long, and total ROH categories to dissect their differential impacts on genomic structure.

### Genomic inbreeding coefficients

To quantify inbreeding levels, four genomic inbreeding coefficients were calculated: runs of homozygosity-based (*F*_ROH_), excess of homozygosity-based (*F*_HOM_), genomic relationship matrix-based (*F*_GRM_), and unified inbreeding coefficient based on uniting gametes (*F*_UNI_). *F*_ROH_ was calculated based on the ROH results detected by PLINK v1.9, by dividing the total length of ROH on the autosomes by the total length of the autosomes [28]. *F*_HOM_ was estimated using the "--het" command in PLINK v1.9. *F*_GRM_ and *F*_UNI_ were computed with the "--ibc" command in GCTA v1.94.1 [39].

### Environmental data processing and statistical analysis

Environmental variables, including annual mean temperature (Bio1) and mean temperature of the warmest quarter (Bio10) from WorldClim v2.1 for the period 1970–2000 at spatial resolution of 30 s [40], and relative humidity (REH) from the NOAA Physical Sciences Laboratory for the period 1991–2020 at a resolution of 0.5, were extracted for each breed's geographic origin using the raster package in R v4.1.2 [41, 42].

To systematically investigate genetic adaptations to cold and heat stress, we classified cattle breeds into four distinct climatic groups based on Bio1, Bio10 and REH values at their geographic origins. We defined Hot climates as those with a Bio10 value greater than the global median for all breeds. Within the Hot group, we further distinguished between Arid-heat adapted (REH below the global median) and Humid-heat adapted (REH above the global median) breeds (Table S1). For cold tolerance, breeds with the lowest Bio1 values were defined as cold-adapted groups (Table S1).

Observed (Ho) and expected (He) heterozygosity were calculated in PLINK v1.9 [28]. Spearman’s rank correlation tests were conducted in R v4.1.2 to evaluate relationships between *F*_ROH_ and Ho/He, and between different inbreeding coefficients, the cumulative length and count across ROH categories, and among ROH category lengths, as well as between ROH allele frequency and environmental factors [41].

### ROH hotspot identification

ROH hotspots were identified across different geographic regions and trait-specific groups (meat, dairy, cold tolerance, heat tolerance) using the --homozyg-group command in PLINK v1.9 [35]. To identify statistically significant ROH hotspots, we performed 10,000 permutations under the null hypothesis of uniform random ROH distribution across the genome [43, 44]. For each group, ROH segments per individual were randomly redistributed within chromosomes while preserving original lengths and counts. To control for the family-wise error rate (FWER), we utilized the IRanges package to compute genome-wide coverage and recorded the maximum SNP-wise frequency per permutation to generate the null distribution [45]. The 99^th^ percentile of this distribution served as the genome-wide significance threshold (empirical *P* < 0.01). Adjacent significant SNPs were merged into ROH hotspots.

### Selection signature analyses

To provide complementary evidence for the evolutionary forces underlying ROH hotspots, we performed three within-population selection scans, including integrated haplotype score (*iHS*), composite likelihood ratio (CLR), and nucleotide diversity (*π*), across geographic and trait-specific groups. The *iHS* statistics were calculated using Selscan v1.3.0 with default parameters, except that the maximum gap was set to 800,000 bp [26]. *iHS* values were normalized using the norm program implemented in Selscan. The proportion of SNPs with |*iHS*| ≥ 2 was computed in 50 kb sliding windows (20 kb step), excluding windows with fewer than 10 SNPs. CLR values were estimated at 1 kb grid intervals using SweeD v3.3.2. To define candidate regions, the maximum CLR value within each 50 kb sliding window (20 kb step) was used as the test statistic. *π* values were calculated using VCFtools within the same sliding window parameters.

For trait-specific selection signatures, we further implemented inter-population tests using cross-population extended haplotype homozygosity (XP-EHH) and the fixation index (*F*_ST_). Five comparative analyses were performed to identify variants underlying growth, milk production, cold tolerance, and heat tolerance (arid-heat and humid-heat): (1) fast-growth European breeds versus unimproved taurine-ancestry Chinese indigenous cattle; (2) specialized dairy versus neighboring beef breeds; (3) high-latitude cold-tolerant breeds versus tropical lineages (defined by Bio1); (4) arid-heat tolerance breeds versus humid-cold tolerance breeds; and (5) humid-heat tolerance breeds versus arid-cold tolerance breeds (Table S1). XP-EHH values were calculated and normalized for 50 kb sliding windows (20 kb step) using Selscan v1.3.0, while Weir and Cockerham’s *F*_ST_ values were estimated using VCFtools with the same sliding window.

For all selection scans, nominal *P*-values for each window were obtained using Z-tests, and windows with empirical *P* < 0.01 were retained as putative selection signals. An ROH hotspot was considered to be supported by selection if it overlapped with a putative genomic region identified by at least one of these selection scans.

### Haplotype distribution visualizations

To elucidate the differences in genotype patterns within ROH hotspots across different population types, we extracted SNPs in these ROH regions from phased SNP data and used the heatmap R package to depict specific genotype patterns.

### Functional annotation analysis

Genes located within ROH hotspots were annotated using the ARS-UCD1.2 reference annotation. The variants in ROH were annotated with ANNOVAR v.2016-02-01 [46]. Gene Ontology (GO) and Kyoto Encyclopedia of Genes and Genomes (KEGG) enrichment analyses were performed using the KOBAS v3.0, with a significance threshold of *P* ≤ 0.05 for enriched terms and pathways. Quantitative trait loci (QTL) overlapping ROH hotspots were identified using CattleQTLdb (Release 57) to link homozygosity to economically and adaptively relevant traits [47].

## Results

### Geographical landscape of ROH

Across global samples, we detected 235,395 ROH segments totaling 313,970 Mb. Using a three-component Gaussian mixture model, ROH were categorized into short (< 756.96 kb), medium (756.96–1,924.89 kb), and long (> 1,924.89 kb) classes (Fig. S1). Short ROH predominated, accounting for 53.62% of the sum ROH count, whereas long ROH were the rarest (13.47%). Sensitivity analyses indicated that ROH detection was primarily sensitive to the number of allowed heterozygous genotypes and the minimum segment length (Fig. S2). Specifically, a plateau effect was observed when the allowed heterozygous SNP count increased from three to five, suggesting that our base setting of three SNPs effectively balanced the tolerance for WGS sequencing errors with detection sensitivity. The 500 kb minimum length threshold was identified as a key inflection point where mean *F*_ROH_ and the average ROH count transition from a disproportionate increase to a stable phase. The high stability of breed rankings across varying parameters reinforced the reliability of our cross-population comparisons.

**Fig. 1:**
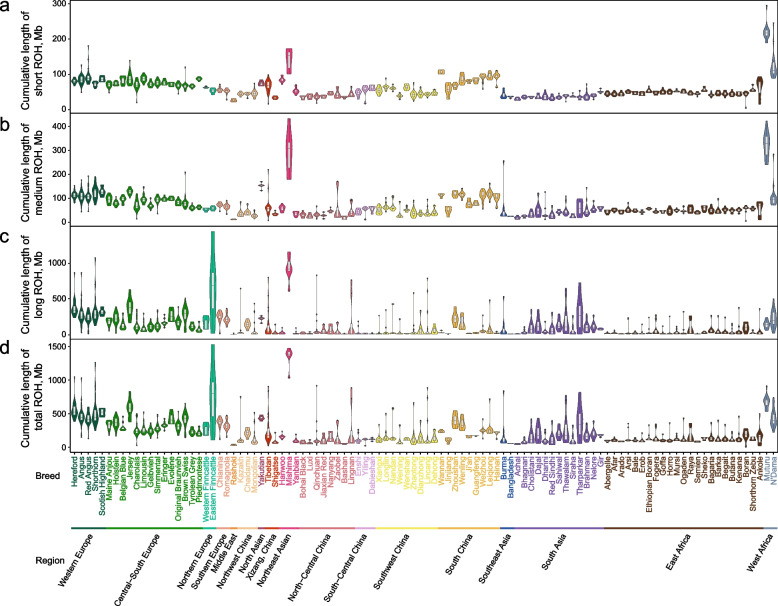
Landscape of individual ROH cumulative length across four length categories in 102 cattle breeds. **a** short (< 756.96 kb), **b** medium (756.96–1,924.89 kb), **c** long (> 1,924.89 kb), and **d** total length

The geographical distribution of ROH burden revealed distinct patterns (Fig. 1, Fig. S3). European breeds, especially those from Western Europe, exhibited elevated ROH across all categories compared to other regions (Wilcoxon rank-sum test, *P* < 3.51e-30), consistent with their extensive history of systematic breeding (Fig. 1). Eastern Finncattle from Northern Europe had exceptionally high long-ROH burden relative to other global breeds (*P* = 0.01) while possessing relatively low short- and medium-ROH levels, suggesting recent inbreeding. West African taurine, including Muturu and N’Dama cattle, showed significantly high short (*P* = 3.48e-21) and medium (*P* = 1.40e-13) ROH. Japanese Mishima cattle had consistently high autozygosity across all categories compared to other breeds (*P* < 1.22e-5), highlighting the genetic consequences of geographic isolation and subsequent inbreeding. Conversely, most Chinese and East African breeds exhibited lower ROH levels than European breeds (all categories, *P* < 4.57e-78). Notably, southern Chinese indicine had a significantly higher short-ROH burden than South Asian indicine from the indicine domestication center (*P* = 2.39e-30).

### Population structure inferred from ROH

To dissect the role of ROH in shaping cattle population structure, we first established a benchmark using whole-genome SNP variation. PCA clearly separated taurine and indicine lineages along PC1, with admixed breeds occupying intermediate positions (Fig. S4). PC2 distinguished indicine populations from Africa, South Asia, and South China, and segregated West African taurine from other taurine groups. Admixture analysis at K = 2 partitioned taurine-indicine ancestries (Fig. S5). At K = 7, combined with geographical location and ancestral origin [1, 3], seven major ancestral components were identified in global cattle: Western European taurine, Central-Southern European (Eurasian) taurine, East Asian taurine, Chinese indicine, South Asian indicine, African indicine, and African taurine.

**Fig. 2:**
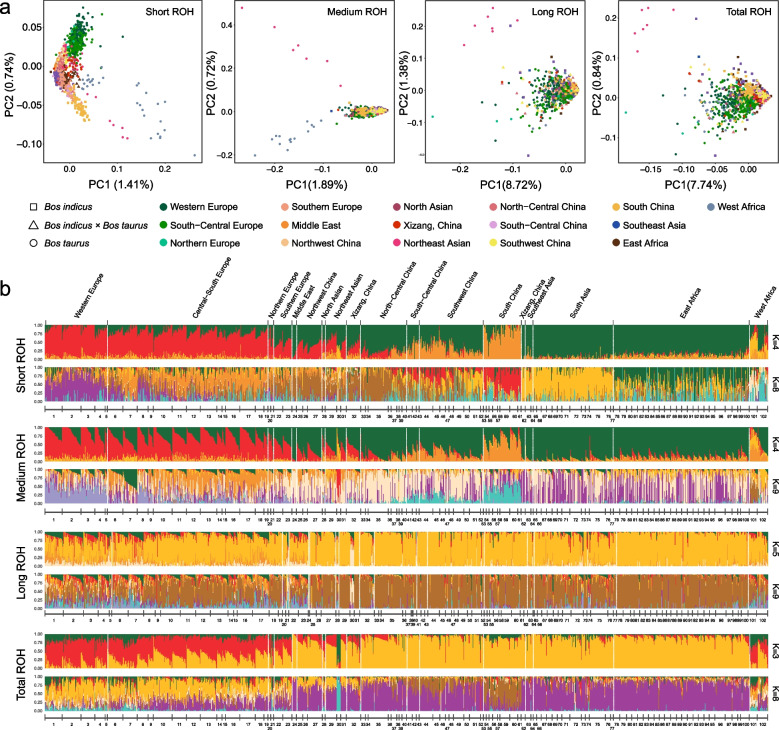
ROH-based population structure analysis, including principal component (PCA) and admixture analysis, for 1,265 samples from 102 cattle populations worldwide.** a** Short, medium, long, and total ROH PCA plots. **b** Short, medium, long, and total ROH admixture analysis plots. The numbers listed in the figure represent (1) Hereford, (2) Angus, (3) Red Angus, (4) Shorthorn, (5) Scotish Highland, (6) Holstein, (7) Jersey, (8) Maine Anjou, (9) Belgian Blue, (10) Charolais, (11) Limousin, (12) Gelbvieh, (13) Simmental, (14) Eringer, (15) Evolène, (16) Original Braunvieh, (17) Brown Swiss, (18) Tyrolean Grey, (19) Piedmontese, (20) Western Finncattle, (21) Eastern Finncattle, (22) Chianina, (23) Romagnola, (24) Rashoki, (25) Kazakh, (26) Chaidamu, (27) Mongolian, (28) Yakutian, (29) Hanwoo, (30) Mishima, (31) Yanbian, (32) Tibetan, (33) Bohai Black, (34) Luxi, (35) Qinchuan, (36) Jiaxian Red, (37) Nanyang, (38) Zaobei, (39) Bashan, (40) Lingnan, (41) Enshi, (42) Yiling, (43) Dabieshan, (44) Xiangxi, (45) Longlin, (46) Nandan, (47) Weining, (48) Wenshan, (49) Zhaotong, (50) Dianzhong, (51) Lincang, (52) Dehong, (53) Wannan, (54) Jinjiang, (55) Zhoushan, (56) Wenling, (57) Ji'an, (58) Guangfeng, (59) Weizhou, (60) Leiqiong, (61) Hainan, (62) Shigatse, (63) Burma, (64) Bangladesh, (65) Achai, (66) Bhagnari, (67) Cholistani, (68) Dajal, (69) Dhanni, (70) Red Sindhi, (71) Sahiwal, (72) Thawalam, (73) Siraha, (74) Tharparkar, (75) Brahman, (76) Nelore, (77) Gir, (78) Abergelle, (79) Afar, (80) Arado, (81) Arsi, (82) Bale, (83) Erob, (84) Ethiopian Boran, (85) Fogera, (86) Goffa, (87) Horro, (88) Mursi, (89) Ogaden, (90) Raya, (91) Semien, (92) Sheko, (93) Bagaria, (94) Barka, (95) Begait, (96) Butana, (97) Kenana, (98) Boran, (99) Shorthorn Zebu, (100) Ankole, (101) Muturu, (102) N'Dama

We then conducted population structure analyses using ROH presence or absence. Short ROH-based PCA primarily separated taurine-indicine along PC2 and endangered breeds (Muturu and Mishima) with other breeds along PC1 (Fig. 2a, Fig. S6). Long ROH-based PCA clustered most cattle, with PC1 differentiating European breeds, Northeast Asian Mishima, and specific South Asian (Tharparkar and Thawalam) cattle from others. Medium ROH-based PCA recapitulated the patterns observed in short ROH across the first three dimensions. Total ROH-based PCA combined effects, highlighting the separation of European, West African, specific South Asian, and Northeast Asian (Mishima cattle) populations. For comparison, PCA using SNPs within ROH regions recapitulated the whole-genome structure, confirming that the unique patterns from ROH-based PCA are due to the sharing of homozygous segments rather than just allele frequency differences (Fig. S7).

ROH-based admixture analysis complemented PCA findings. For short ROH, at K = 2, Muturu cattle formed a distinct ancestry compared to other global cattle (Fig. 2b, Fig. S8); at K = 4, South Chinese indicine formed a distinct ancestry; at K = 8, Western Europe, Central-South/Northern Europe, Northeast Asia, South China, South Asia, East Africa, and West Africa each exhibited distinct ROH ancestries, with Jersey cows splitting at K = 9. For long ROH, K = 2 isolated Mishima (Fig. 2b, Fig. S9); at K = 5, besides Mishima cattle, the European breeds and some individuals of the South Asian Tharparkar and Thawalam shared a distinct ancestry that differed from that of cattle from other regions, which likely highlights the genomic impact of recent consanguinity. Medium ROH admixture analyses at K = 2 isolated Mishima and Muturu contrasted with global cattle (Fig. 2b, Fig. S10); K = 3 split European from non-European (excluding Mishima); K = 4 flagged South Chinese indicine; K = 9, defined unique ancestries for Western Europe, Central-South Europe, Northeast Asia, South China, East Africa, and West Africa, as well as distinguishes Jersey cattle. Total ROH admixture revealed that, at K = 3, separated European versus non-European ancestries (excluding Mishima), with Western European and two dairy breeds (Holstein and Jersey) exhibiting different ancestry compositions from other Europeans (Fig. 2b, Fig. S11); at K = 8, South China indicine showed unique compositions. These length-specific patterns underscore the utility of ROH for disentangling both deep demographic history and recent selection or inbreeding events.

### Length-count correlations across ROH classes resolve demographic signals in global cattle

To further investigate demographic history (Fig. 1, Fig. S3), we performed pairwise comparisons between the cumulative length and count per individual. Across the 1,265 unrelated samples, these two metrics were strongly correlated within the short, medium, and long categories (Global *r* ≥ 0.98, *P* < 0.001; Fig. S12). While this strong correlation held across most regions, the total ROH category exhibited greater regional variability, with lower correlations in South China (*r* = 0.64), Western Europe (*r* = 0.66), and Central-South Europe (*r* = 0.67). This pattern likely reflects the confounding effects of intensive artificial selection and fluctuating effective population sizes on overall genomic architecture. Furthermore, in West African cattle, the correlation for long ROH (*r* = 0.59) was markedly lower than for shorter segments (*r* > 0.99), suggesting distinct recent demographic or inbreeding pressures.

**Fig. 3:**
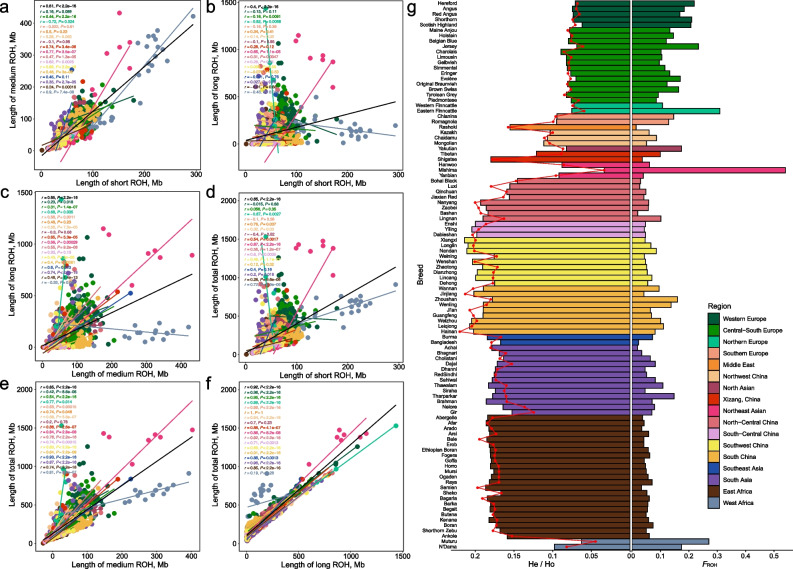
The length class divergent pattern of ROH and its shaping of global cattle inbreeding patterns and genomic diversity landscape.** a** Correlation between the individual short and medium ROH lengths. **b** Correlation between the individual short and long ROH lengths. **c** Correlation between individual short and total ROH length. **d** Correlation between individual medium and long ROH lengths. **e** Correlation between individual medium and total ROH length. **f** Correlation between individual of long and total ROH lengths. **g** Population-average expected heterozygosity (He, bars), observed heterozygosity (Ho, points and line), and ROH-based inbreeding coefficient (*F*_ROH_, bars) across 102 global breeds

Inter-category length correlations further decoupled ancient demographic signals from recent inbreeding and selection (Fig. 3a–f). Globally, cumulative lengths of short and medium ROH were highly associated (*r* = 0.81, *P* < 0.001), whereas both exhibited significantly weaker correlations with long ROH (*r* = 0.40 and 0.65, respectively). This divergence is consistent with observations in human populations, where long ROH reflect recent inbreeding and short/medium capture population-level LD patterns across varying evolutionary timescales [8]. Medium and long ROH were the dominant contributors to the total ROH burden, showing strong correlations with total length (*r* = 0.85 and 0.92, respectively), while short ROH showed a relatively weak association with the total ROH (*r* = 0.65). Regionally, significant positive associations between short and medium ROH were observed in 11 of the 17 groups, whereas the correlation between short and long ROH remained significant in only four regions. Notably, Northern Europeans had significant negative short-medium (*r* = −0.72) and short-long (*r* = −0.82) correlations, implying divergent pressures between deep ancestral diversity and recent breeding-induced autozygosity. Medium-long ROH correlations were significant in 13 regions (*P* < 0.05), peaking in South Asia (*r* = 0.74) and Northern Europe (*r* = 0.68). Furthermore, total ROH burden was significantly correlated with long ROH across groups, except for West African cattle (*r* = 0.19, *P* = 0.29). These variations highlight region-specific breeding and demographic histories shaping ROH architecture.

### Genomic inbreeding coefficient across global cattle

Genomic inbreeding was assessed using *F*_ROH_, *F*_HOM_, *F*_GRM_, and *F*_UNI_ (Table S2). The global *F*_ROH_ average was 0.099 (ranging from 0.014 to 0.540), with the highest values observed in Mishima (0.540), Eastern Finncattle (0.309), and Muturu (0.270) cattle (Fig. 3g). Significant regional variation was observed, with West African cattle displaying the highest regional *F*_ROH_ (0.217), reflecting notable inbreeding levels. European cattle herds generally displayed high *F*_ROH_ values, including Western Europe (0.196), Central-South Europe (0.131), and Northern Europe (0.209), reflecting intensive breeding. Whereas lower *F*_ROH_ values were observed in East African (0.052), the Middle East (0.014), and several Chinese regions (e.g., North-Central China, *F*_ROH_ = 0.050; Southwest China, *F*_ROH_ = 0.058), reflecting low levels of inbreeding and selection.

Other inbreeding coefficients exhibited considerable variability across breeds, with patterns not always aligning with *F*_ROH_ (Table S2). *F*_HOM_ ranged from −0.181 (Hainan, South China) to 0.819 (Mishima, Northeast Asia), with a global mean of 0.291. The highest *F*_HOM_ values were observed in Mishima (0.819) and Muturu (0.760), consistent with their elevated *F*_ROH_. Negative average *F*_HOM_ values were prevalent in regions such as South China (−0.074), South-Central China (−0.036) and Southwest China (−0.005), likely arising from allele frequency biases or recent admixture. *F*_GRM_ showed substantial variation, ranging from −0.335 (Maine Anjou, Central-South Europe) to 2.138 (Leiqiong, South China), with an overall mean of 0.223, and the highest regional averages in South China (1.987) and South-Central China (1.119). *F*_UNI_ ranged from 0.053 (Rashoki, Middle East) to 0.647 (Wenling, South China), with a global mean of 0.223, and the elevated averages in South China (0.561) and South Asia (0.357). These patterns were similar to prior studies in sheep and pig, where *F*_ROH_ effectively captures inbreeding, unlike *F*_HOM_ and *F*_GRM_ are more sensitive to allele frequencies, often yielding negative estimates in outbred or admixed populations [18, 19, 48].

Spearman’s rank correlations among inbreeding coefficients are summarized in Table S3. Globally, *F*_ROH_ is highly correlated with *F*_HOM_ (*r* = 0.691, *P* < 0.001) and weakly with *F*_UNI_ (*r* = 0.312, *P* < 0.001), but negatively with *F*_GRM_ (*r* = −0.377, *P* < 0.001). Regionally, European breeds showed consistently high correlations across all metrics, while Asian and African regions displayed greater variability. South China displayed strong *F*_ROH_-*F*_HOM_ correlation (*r* = 0.752) but weaker *F*_ROH_-*F*_GRM_ correlation (*r* = 0.320) and *F*_HOM_-*F*_GRM_ correlation (*r* = 0.069). West Africa mirrored this, with *F*_ROH_-*F*_HOM_ (*r* = 0.771), *F*_ROH_-*F*_GRM_ (*r* = 0.402), and *F*_HOM_-*F*_GRM_ (*r* = 0.032).

To evaluate the impact of inbreeding on genetic diversity, average observed heterozygosity (Ho) and expected heterozygosity (He) were calculated (Fig. 3g). Most breeds exhibited Ho and He values below 0.2, with Ho slightly lower than He. *F*_ROH_ showed negative correlations with both Ho (*r* = −0.589) and He (*r* = −0.492, Fig. S13), with cattle from North-Central China, South-Central China, South China, South Asia, and East Africa showing higher heterozygosity and lower *F*_ROH_, whereas European and West African breeds generally displayed the opposite trend. These findings collectively underscore how breeding and demography have shaped autozygosity and genetic diversity in cattle worldwide.

### Regional ROH hotspots reflect local adaptation and demographic legacies

Genomic regions with the highest frequency of ROH within populations are termed ROH hotspots [18, 49]. By employing a permutation test (empirical *P* < 0.01), we determined group-specific relative frequency thresholds to identify hotspots that were significant beyond random expectation, based on the null distribution reflecting each group's intrinsic background homozygosity (Table S4). We observed that the relative frequency threshold was significantly inversely correlated with sample size across 17 regions (*r* = −0.871, *P* < 0.001), while the number of identified significant SNP hotspots was positively associated with sample size (*r* = 0.897, *P* < 0.001). This relationship reflects higher statistical power in larger groups while ensuring conservative control of false positives in smaller or inbred populations.

Overlap analysis of ROH hotspot SNPs revealed that geographically proximate or ancestrally related regions share a substantial number of these loci (Fig. S14). For instance, Western and Central-South Europe shared 169,465 loci, and South China and Southwest China shared 46,277 loci. Additionally, East Africa and South Asia shared 58,105 loci, consistent with previous evidence for the historical migration of African indicine cattle originating from South Asia [1]. Merging adjacent SNPs for gene annotation yielded 29–1,518 genes across the 17 regions (Table S5, Fig. S15). Notably, a conserved ROH hotspot on BTA7 (50.04–50.84 Mb) was detected in 15 regions (excluding Northern Europe and North Asia), harboring 14 genes involved in muscle development (*CTNNA1*, *MATR3*) [50, 51], host immunity (*MATR3*, *MZB1*, and *STING1*) [52], fertility (*PAIP2*, *LRRTM2*) [53, 54]. This hotspot, consistently identified in prior ROH studies [13, 55], likely highlights a functionally important homozygous region in the bovine genome.

To evaluate whether ROH hotspots are consistent with signals of positive selection, we cross-validated them with three complementary within-population selection scans (*iHS*, CLR, and *π*). On average, 57.90% of ROH hotspot SNPs overlapped with at least one selection scan, ranging from 28.39% in North Asia to 90.46% in Northern Europe. SNPs supported by at least two selection methods ranged from 2.66% in North Asia to 54.30% in Southeast Asia (Table S4). This multi-method convergence suggests that a substantial portion of the ROH hotspots are likely driven by positive selection, while demographic effects (e.g., bottlenecks, drift, population size changes, migration, and admixture) may remain an important contributing factor, particularly in regions with lower overlap.

Functional enrichment analysis of all ROH hotspot genes revealed conserved functions across regions, including cell cycle, ubiquitin-mediated proteolysis, endoplasmic reticulum protein processing, and RNA degradation (Table S6). Beyond these, region-specific enrichments mirrored local adaptive and breeding pressures (Table S6). European breeds showed broad enrichment in cell division, actomyosin structure organization, thermogenesis, lipid metabolism, and cancer pathways, consistent with intensive artificial selection for growth and meat quality (Table S6). Sub-regional patterns emphasized skeletal muscle differentiation in Western, Central-South, and Southern European cattle, alongside signals for antioxidant and erythrocyte differentiation in Northern Europe (Table S6). Middle Eastern cattle showed enrichment in ion and sialic acid transport. Similarly, Northwest China displayed signals for UV response and aldosterone-regulated sodium reabsorption, reflecting local challenges of high radiation and aridity. North Asian breeds emphasized fatty acid synthesis and metabolism, supporting cold adaptation. Tibetan cattle showed enrichments in p53 signaling, DNA repair, immune response, and apoptosis, consistent with high-altitude hypoxia adaptation. North-Central and South-Central Chinese cattle displayed distinct features in immune and metabolic regulation. South Chinese cattle showed enrichment in keratinocyte development, skin barrier, and arachidonic acid metabolism, likely adaptations to humid-heat climates. Southeast and South Asian cattle focused lipid metabolism, apoptosis, cell adhesion, and immune regulation, supporting heat tolerance. In Africa, eastern breeds enriched mitochondrial ATP synthesis, water absorption, calcium ion response, and immune regulation, whereas West African cattle, known for their small size and trypanosomiasis resistance, showed strong signals in bacterial invasion of epithelial cells, aldosterone regulation of sodium reabsorption, and the Hippo and Ras signaling pathways, consistent with tropical adaptation and disease resistance.

To further evaluate the contribution of selection to these region-specific patterns, enrichment analyses were repeated using only ROH genes supported by at least one independent selection scan. This subset enrichment largely recapitulated and reinforced the primary findings, such as thermogenesis and lipid storage in Northern Europe, hypoxia/energy pathways in Xizang, and skin barrier functions and arachidonic acid metabolism in South China (Table S7). Conversely, many region-specific functions were weakened or absent in low-validation regions, such as North Asia. These results further support that ROH hotspots, particularly those co-validated by selection scans, effectively capture genomic variations shaped by natural and artificial selection.

### ROH variants underlying key economic and adaptive traits

To identify trait-specific ROH variants, we categorized breeds by production type or adaptive trait to enhance the frequency of key alleles. To validate that these ROH hotspots reflect selective pressures, we cross-validated them with five complementary selection signatures (*iHS*, CLR, *π*, *F*_ST_, and XP-EHH). ROH hotspots with selection-validated were given particular emphasis.

**Fig. 4:**
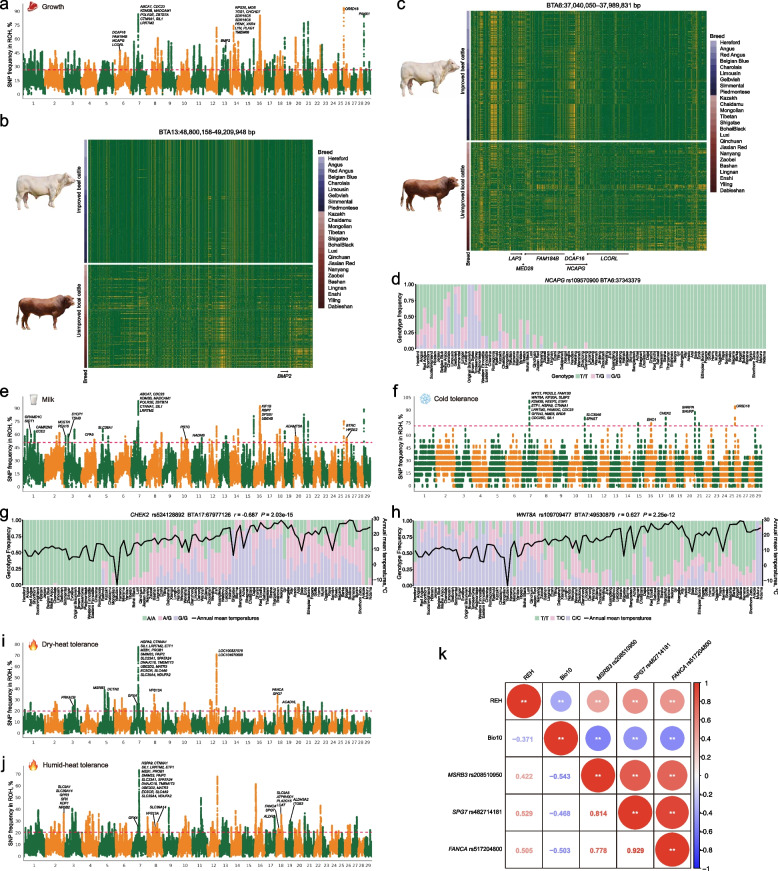
Identification of ROH hotspots associated with key economic and adaptive traits.** a** Manhattan plot of genome-wide ROH hotspots for growth traits. **b** Haplotype structure in the BTA13: 48,800,158–49,209,948 interval in improved beef cattle (intensively selected European breeds: Hereford, Angus, Red Angus, Belgian Blue, Charolais, Limousin, Gelbvieh, Simmental, and Piedmontese) versus unimproved local cattle (indigenous Chinese breeds with taurine ancestry lacking systematic breeding programs: Kazakh, Chaidamu, Mongolian, Tibetan, Shigatse, Bohai Black, Luxi, Qinchuan, Jiaxian Red, Nanyang, Zaobei, Bashan, Lingnan, Enshi, Yiling, and Dabieshan). **c** Haplotype structure in the BTA6: 37,026,816–37,849,977 bp interval in improved beef cattle versus unimproved local cattle. **d** Global allele frequency distribution of *NCAPG* rs109570900 across 102 cattle breeds. **e** Manhattan plot of genome-wide ROH hotspots for milk production traits. **f** Manhattan plot of genome-wide ROH hotspots for cold tolerance traits. **g** Genotype frequencies of missense mutations in *CHEK2* (rs524128892) across global cattle populations, plotted against annual mean temperature.** h** Genotype frequencies of the *WNT8A* missense mutation rs109709477 across global cattle populations, plotted against annual mean temperature. **i** Manhattan plot of genome-wide ROH hotspots in cattle adapted to hot-dry environments.** j** Manhattan plot of genome-wide ROH hotspots in cattle adapted to hot-humid environments. **k** Spearman correlation analysis of missense mutations in heat-tolerance genes with mean temperature of the warmest quarter (Bio10) and relative humidity (REH). * *P* < 0.05, ** *P* < 0.01

For growth traits, we analyzed nine major beef cattle with close ancestry, including Angus, Red Angus, Belgian Blue, Charolais, Gelbvieh, Hereford, Limousin, Simmental, and Piedmontese. Merging adjacent SNPs yielded 8,751 ROH segments harboring 1,529 genes (Fig. 4a, Table S8), enriched in mTOR signaling, cell growth, and skeletal muscle differentiation (Table S9). The most prominent ROH was located within BTA26: 77,953–638,753 bp, harboring *OR5D18*, an olfactory receptor gene potentially linked to feed intake [56]. Cross-validation revealed that 48.89% (involving 909 genes) and 23.25% (involving 448 genes) of high-frequency ROH SNPs were supported by at least one and two selection statistics, respectively. Within selection-supported hotspots, the BTA13: 48,800,158–49,209,948 bp region contained *BMP2* gene, associated with body size, skeletal, and muscle development across cattle [26, 57], pigs [58], sheep [59], and chicken [60], and showed haplotype differentiation in populations with differential growth rates (Fig. 4b). Another hotspot on BTA14: 22,720,334–23,689,976 bp encompassed a gene cluster (*RPS20*, *MOS*, *TGS1*, *CHCHD7*, *SDR16C5*, *SDR16C6*, *PENK*, *XKR4*, *LYN*, *PLAG1*, and *TMEM68*), linked to stature, body weight, carcass weight, and feed intake [61]. A significant ROH hotspot on BTA6: 37,040,050–37,989,831 bp, covered multiple growth-related QTLs [62], containing *DCAF16*, *FAM184B*, *NCAPG*, and *LCORL*, with distinct homozygous haplotypes between fast- and slow-growth cattle (Fig. 4c). Two missense mutations in *NCAPG* (rs109570900, c.T1161G, p.I387M; and rs110251642, c.C2464A, p. L822M) displayed marked allele frequency differences between improved beef cattle and unimproved local cattle (Fig. 4d, Fig. S16).

For milk production traits, we focused on specialized dairy breeds, including Holstein and Jersey cows, and identified 1,311 ROH hotspots containing 553 genes (Fig. 4e, Table S8). Enrichment analysis highlighted functions in protein synthesis, cell proliferation, cancer signaling, and endocrine regulation (FDR < 0.05), supporting efficient mammary gland function and lactation metabolism (Table S9). Notably, 72.17% of these SNPs were supported by at least one selection signature, encompassing 446 genes. According to the CattleQTLdb database, genes including *ABCA7*, *ADAMTS6*, *BTRC*, *DIDO1*, *FSTL4*, *GRAMD1C*, *HADHB*, *HPSE2*, *KDM3B*, *MADCAM1*, *MASP2*, *NCSTN*, *NEGR1*, *PRTG*, *SPSB1*, *SYCP1*, *TSHB*, and *ZBTB7A*, have been previously associated with milk yield and milk composition (Table S8) [63–66].

To investigate ROH-associated functional variants underlying cold tolerance, we focused on Yakutian, Eastern Finncattle, and Western Finncattle breeds from high-latitude regions of North Asia and Northern Europe, where annual mean temperatures were lowest worldwide. Despite the conservative nature of the permutation test (*P* < 0.01) necessitated by the limited sample size, 99.40% of the 31,025 identified high-frequency SNPs were cross-validated by selection signatures. Merging adjacent validated SNPs yielded 91 high‑frequency ROH hotspots encompassing 43 genes (Table S8). These candidates are implicated in essential adaptive processes, including DNA damage response (*CHEK2*) [67], beige fat regulation (*EGR1*) [68], and metabolic adaptation to cold stress (*SLC30A6*) [69], glycolytic enzyme (*ENO1*) [70], and lipid metabolism (*SPAST*) [71]. Spearman correlation analysis revealed negative correlation between Bio1 and the frequency of two highly linked (*r*^2^ = 0.956) missense variants in the *CHEK2* (rs524128892, c.T1541C, p.L514P, *r* = −0.687; and rs520226567, c.A1307G, p.Y436C, *r* = −0.688) (Fig. 4g, Fig. S17). Furthermore, the Wnt signaling pathway plays a key role in preadipocyte proliferation and differentiation [72], which is critical for thermogenic adaptation. A missense variant frequency in *WNT8A* (rs109709477, c.T1033C, p.S345P, *r* = 0.627) showed a significant positive association with annual mean temperature (Fig. 4g). These findings underscore the functional importance of ROH‑associated variants in thermogenesis, energy homeostasis, and cellular integrity under cold stress.

High temperatures can cause heat stress in cattle, with heat adaptation gaining increasing importance amid climate change. To elucidate ROH-associated functional variants underlying heat tolerance in cattle, we classified breeds as arid-heat or humid-heat tolerant based on Bio10 and REH values (Fig. S18). For arid-heat tolerance, 12 cattle breeds from South Asia and Africa (Table S1) yielded 987 ROH segments with 432 genes (Fig. 4i, Table S8), enriched for oxidative phosphorylation, fatty acid metabolism, DNA damage repair, sodium ion transport, tissue homeostasis, and cellular stress responses (Table S9). For humid-heat tolerance, 15 breeds from South, Southwest, and Central China (Table S1) generated 3,582 ROH segments with 1,155 genes (Fig. 4j, Table S8), enriched for skin barrier establishment, keratinocyte development, lipid/amino acid metabolism, ferroptosis regulation, and pathogen defense (Table S9). Approximately 58.05% and 60.33% of high-frequency SNPs within arid- and humid-heat ROH hotspots, respectively, overlapped with other putative selection signatures. Among these selection-driven candidates, 168 genes were shared between both heat-adapted groups, including those associated with heat stress resistance (*VPS13A* [73]), heat shock response (*DNAJC18* [1, 74], *HSPA9* [75]), oxidative stress response (*GPX4*, *SLC35A4*, *SLC23A1* [76, 77]), DNA repair (*FANCA* [78]), vasodilation for heat dissipation (*ECSCR* [79]), degrades misfolded proteins (*SPG7* [80]), and immune response (*TMEM173*, *MZB1*, *SIL1*). Missense allele frequencies in *FANCA* (rs517204800, c.C3659G, p.P1220R, *r* = 0.510 in REH and −0.507 in Bio10) and *SPG7* (rs482714181, c.C1384T, p.R462W, *r* = 0.529 in REH and −0.468 in Bio10) were significantly correlated with climatic variables (*P* < 0.01). Furthermore, arid-heat-specific genes involved in vasopressin-regulated water reabsorption (*DCTN2*, *PRKACB*), melanogenesis (*DVL2*, *MC1R*), fatty acid metabolism (*ACADVL*), and heat stress resistance (*MSRB3* [81]) for water retention, thermoregulation, and radiation protection, with a missense mutation in *MSRB3* (rs208510950, c.G238A, p.A80T, *r* = 0.422 in REH and −0.543 in Bio10) significantly correlated with REH and Bio10 (*P* < 0.01) (Fig. 4k, Fig. S19). Conversely, humid-heat-specific gene functions encompassed brown adipose thermogenesis (*GPR3* [82]), skin barrier formation (*SFN*, *KDF1* [83, 84]), ion transport (*SLC9A5*, *SLC9A1*, *SLC39A14* [85, 86]), and lipid regulation (*NR0B2* [87], *ALDH2* [87], *LCAT* [88]) for against pathogen pressure and thermoregulatory in humid environments.

## Discussion

Inherited identical haplotypes from common ancestors lead to ROH, a ubiquitous feature in livestock genomes [9, 89]. By analyzing a global dataset of cattle herds, this study systematically deciphers the role of ROH in elucidating demographic history, quantifying inbreeding, and pinpointing hotspots underlying key economic and adaptive traits. We found that ROH length classes can be used to dissect evolutionary forces temporally, distinguishing ancient linkage disequilibrium from recent selective pressures and inbreeding events. These patterns reveal divergent population histories and highlight ROH hotspots as functionally important markers for economic traits and environmental resilience. These findings extend prior array-limited analyses [7, 9–11, 13], offering finer-resolution insights into cattle genomic dynamics to inform sustainable breeding.

A common challenge in ROH studies is bias from arbitrary parameter settings. Unlike traditional SNP arrays, WGS data offer superior marker density but are susceptible to fragmentation from sequencing artifacts. Sensitivity analyses supported the robustness of the observed ROH patterns, where base parameters consistently fell within stable ranges for key metrics (Fig. S2). This reinforces the reliability of our cross-population comparisons.

The geographic distribution of ROH underscores the combined impact of both historical demography and contemporary management. European breeds exhibited elevated homozygosity across all ROH categories, consistent with intensive selective breeding that accelerates the fixation of favorable alleles [90, 91]. Conversely, island isolated populations like Japanese Mishima cattle exhibit extreme burdens, a classic signature of recent inbreeding resulting from geographic bottlenecks. Similarly, West African trypanosomiasis-resistant taurine breeds, including Muturu and N’Dama, showed elevated ROH levels, aligning with population decline due to a small size disadvantage, which makes them less competitive in intensive production systems [92, 93]. In contrast, lower ROH in East African and certain Chinese groups suggests a history of less stringent selection. Interestingly, unlike patterns observed in humans and sheep, where ROH increases with distance from origins [8, 48], this study did not find an obvious gradient in cattle. For instance, several breeds from the Indus Valley domestication center showed elevated medium and long ROH, whereas the Rashoki cattle from neighboring areas of the Fertile Crescent maintained low levels across all categories, though sampling in this region remains limited and warrants future expansion.

ROH-based population structures provide a temporal lens surpassing geographic patterns. Short ROH captured deep domestication signals, such as taurine-indicine divergence and the unique ancestral composition of South Chinese indicine. Long ROH results, conversely, spotlight the effects of recent selection and inbreeding in European cattle. The marked divergence between ROH-based and standard SNP-based structures underscores the unique utility of ROH as identity-by-descent markers [94], offering a complementary perspective on population history.

Notably, South Chinese indicine displayed a high short ROH burden globally and formed a unique ancestry in short-ROH admixture analysis (K = 4–20; Fig. 2b, Fig. S8), a pattern absent in long ROH profiles. We propose that this reflects a unique demographic history rather than recent inbreeding. Previous studies suggested introgression from banteng or gaur contributed to elevated genetic diversity in South Chinese indicine [1, 34]. Our analysis showed that the frequency of ROH, overlapping with introgression fragments in South Chinese cattle, was not high globally (Fig. S20). Paired Wilcoxon signed-rank tests revealed significant short ROH frequency divergence in South Chinese indicine from other regions’ cattle (10/16 comparisons, *P* < 0.05), versus only 4 comparisons for long ROH (Fig. S20). We speculate that ancient introgression events may have subtly altered the genomic ROH architecture, potentially elevating recombination rates and increasing short ROH through subsequent migration bottlenecks. This signal that short ROHs preserve ancient mixing events was further explored in Central China, a typical zone of historical taurine-indicine hybridization and migration (Fig. S5). Across 11 indigenous breeds (*n* = 102) spanning this transition belt, indicine ancestry proportion (Whole-genome SNP admixture K = 2; Fig. S5) was most strongly correlated with cumulative short-ROH length (*r* = 0.516, *P* = 2.78e-8) (Fig. S21).

Pedigree-based inbreeding coefficient estimates were the traditional standard [19]. However, in global-scale comparisons, genomic metrics like *F*_ROH_, *F*_HOM_, *F*_GRM_, and *F*_UNI_ overcome the widespread limitations of absent or incomplete pedigrees and capture historical inbreeding [19, 95]. The correlations between different inbreeding coefficients varied considerably across studies. Our findings show that *F*_ROH_ exhibits moderate to high correlations with *F*_HOM_ across geographic regions, while its correlation with *F*_GRM_ varied significantly across regions, and avoids biologically implausible negative values. However, previous array-based studies reported low to moderate *F*_GRM_-*F*_ROH_ correlations in Italian cattle breeds [96, 97], with medium to high correlations specifically documented in Jersey, Holstein, and Chinese indigenous cattle populations [13, 98, 99]. These discrepant results may stem from differences in sample populations, number of variants, and SNP genotyping platforms [13]. The high *F*_ROH_ in intensively bred or isolated breeds is ​​accompanied by diminished heterozygosity (Ho/He), signaling potential vulnerabilities to inbreeding depression, whereas lower levels in unimproved populations preserve adaptive potential. Furthermore, previous studies have found a strong correlation between ROH-based inbreeding coefficients and pedigree inbreeding coefficients in cattle [7]. This supports the use of ROH-based inbreeding coefficients to monitor and mitigate the loss of genetic diversity in breeding and conservation programs.

A permutation-based approach was employed to identify significant ROH hotspots by randomly redistributing intact ROH segments. This method generates a null distribution reflecting each group's intrinsic background homozygosity, yielding adaptive empirical thresholds (*P* < 0.01) rather than arbitrary fixed cutoffs [9, 11, 18, 19]. Unlike genotype-shuffling permutations [96], this strategy preserves the intrinsic LD structure of the genome by redistributing intact segments. The resulting relative frequency thresholds proved highly adaptive, showing a significant inverse correlation with sample size (*r* = −0.871, *P* < 0.001). For instance, in small or highly inbred populations (e.g., Northern Europe and North Asia), the model generated more conservative thresholds (80%−100%). This adaptive framework effectively suppressed stochastic noise in small cohorts while unlocking high-resolution detection in larger groups, ensuring that identified hotspots are statistically significant.

While ROH hotspots highlight regions of elevated autozygosity accumulated over extended evolutionary timescales, they can result from diverse forces, including selection, demographic bottlenecks, or intensive breeding. The high degree of overlap between ROH hotspots and independent selection signatures suggests many hotspots are likely driven by positive selection, whereas non-validated hotspots may primarily reflect historical demographic legacies. Regional ROH hotspot analysis reveals a blend of widespread purifying selection on conserved functions, alongside region-specific directional adaptation. Notably, the ubiquitous hotspot on BTA7, which harbors genes involved in muscle development (*CTNNA1* [50]) and immunity (*STING1* [52]), was detected in multiple previous ROH analyses of livestock, underscoring its conserved functional importance across species [13, 55, 100].

Trait-specific ROH hotspot analyses further bridged homozygosity to phenotypes. Growth-related hotspots, harboring *BMP2* [26, 57–59], *PLAG1* [61], *NCAPG* [101, 102], and *LCORL* [62] genes linked to body weight, average daily gain, carcass weight, and feed conversion, were supported by multiple selection metrics. Missense variants (rs109570900 and rs110251642) in *NCAPG* exhibited allele frequency differences between fast- and slow-growing breeds (Fig. 4d, Fig. S16), with cattle carrying the 442M allele at *NCAPG* rs109570900 displaying superior feed intake and conversion efficiency [101, 102]. Similarly, dairy-related hotspots highlighted genes supporting lactation metabolism (*ABCA7*, *HADHB*, *KDM3B*, *NEGR1*, etc.), with 72.17% of SNPs cross-validated by selection signatures, underscoring the role of selection in shaping milk production traits. Cold-adaptation hotspots implicated thermogenesis and energy metabolism pathways, with missense variants in *CHEK2* negatively correlated with annual mean temperature (Fig. 4g–h, Fig. S17). Heat tolerance hotspots revealed shared stress genes across arid and humid heat adaptations, including *DNAJC18, HSPA9, SLC4A9,* and *ECSCR*, with missense variants in *FANCA* (rs517204800) [78] and *SPG7* (rs482714181) [80] correlated with climatic variables (Fig. 4k, Fig. S19). The arid-heat climate-specific ROH hotspots further highlight a climate-correlation missense mutation (rs208510950) in *MSRB3* [81]. The finding that these adaptive alleles show divergent frequencies between taurine and indicine lineages is consistent with their distinct domestication origins and subsequent adaptation to contrasting environments [103, 104].

Despite these advances, several limitations remain. The limited sample size in the Fertile Crescent domestication center and its surrounding areas may constrain the resolution of early domestication. While our sample-adaptive permutation test mitigates sample size bias in hotspot detection, its conservative threshold in small populations may lead to the neglect of some important functional variants. Meanwhile, while selection-supported hotspots represent high-confidence candidates, other ROH regions may still reflect adaptive variation shaped by historical demographic factors. Relying on historical breed origins for bioclimatic variables may not fully capture contemporary environmental exposures of widely distributed commercial breeds, which may have been subjected to different selective pressures in their modern production environments. Furthermore, definitively disentangling the relative contributions of selection, inbreeding, and neutral demographic processes to the formation of specific ROH regions remains a fundamental challenge, as these forces can interact and produce convergent genomic patterns. Future studies integrating larger and balanced samples, multi-omics data, and demographic simulations will be crucial for validating causal variants and further disentangling these intertwined factors.

## Conclusions

By analyzing ROH in a global cattle WGS panel, our study underscores that ROH may serve as genomic markers of temporal stratification, dissecting cattle population history dynamics, assessing inbreeding levels, and pinpointing functional variants underlying economic and climate resilience. These findings provide critical insights into the conservation of genetic diversity and precision breeding in cattle.

## Supplementary Information

Additional file 1: Table S1. Overview of sample information and sequencing statistics for the global cattle panel. Table S2. Four inbreeding coefficients of 102 cattle breeds from 17 regions worldwide. Table S3. Spearman correlation between four inbreeding coefficients, including *F*_ROH_, *F*_HOM_, *F*_GRM_, and *F*_UNI_. Table S4. Permutation‑derived ROH hotspot thresholds and cross‑validation with selection signatures across 17 geographic regions. Table S5. Gene annotation of regional ROH hotspots identified via permutation tests (*P* < 0.01). Table S6. Functional enrichment analysis of regional ROH hotspot genes based on KOBAS v3.0 database. Table S7. Functional enrichment analysis of genes within regional ROH hotspots cross-validated by selection scans based on the KOBAS v3.0 database. Table S8. Gene annotation of ROH hotspots associated with cattle production and adaptive traits. Table S9. Functional enrichment analysis of ROH hotspot genes for cattle adaptation and production traits based on KOBAS v3.0 database.

Additional file 2: Fig. S1 Classification of ROH into three size categories using a three-component Gaussian mixture model. Fig. S2 Sensitivity analysis of ROH identification parameters across seven representative cattle breeds with diverse genetic backgrounds. Fig. S3 Landscape of individual ROH cumulative number across four categories in 102 cattle populations. Fig. S4 Principal component analysis based on whole-genome SNP variations. Fig. S5 Admixture analysis for K = 2–20 based on whole-genome SNP variations. Fig. S6 Principal component analysis diagram based on the existence of ROH. Fig. S7 PCA plot based on SNP genotypes within different categories of ROH segments. Fig. S8 Admixture plot for short ROH with K = 2–20. Fig. S9 Admixture plot for long ROH with K = 2–20. Fig. S10 Admixture plot for medium ROH with K = 2–20. Fig. S11 Admixture plot for total ROH with K = 2–20. Fig. S12 Spearman correlation between ROH length and number across four length categories. Fig. S13 Spearman correlation analysis between *F*_ROH_ and observed (Ho) or expected (He) heterozygosity. Fig. S14 UpsetR map of ROH hotspots in cattle genomes from 17 regions. Fig. S15 Manhattan plot of the frequency of SNPs within runs of homozygosity (ROH) across 17 global cattle populations. The dashed line represents the permutation test threshold *P* < 0.01 used to identify ROH hotspots. Fig. S16 Global allele frequency distribution of *NCAPG* rs110251642 associated with growth across 102 cattle breeds. Fig. S17 Global allele frequency distribution of *CHEK2* rs520226567 associated with cold adaptation across 102 cattle breeds. Fig. S18 Climatic niche distribution of cattle breeds based on mean temperature of the warmest quarter (Bio10) and relative humidity (REH). Fig. S19 Environmental correlation analysis of missense variants within selection-validated heat tolerance ROH hotspots. Fig. S20 Paired Wilcoxon signed-rank test for frequency of ROH located within wild bovine-introgressed (banteng or gaur) regions of Southern Chinese indicine cattle from two previous studies. Fig. S21 Scatter plots display Spearman correlations between indicine ancestry proportions (derived from whole-genome SNP Admixture analysis at K = 2) and cumulative lengths of four ROH categories across 125 individuals from 11 cattle breeds in Central China.

## Data Availability

The WGS data were deposited into the NCBI BioProject database under access number PRJNA1252684, accessible at: https://submit.ncbi.nlm.nih.gov/subs/sra/SUB15780934/overview

## Abbreviations

Bio1: Annual mean temperature
Bio10: Mean temperature of the warmest quarter
*F*_ROH_: Inbreeding coefficient based on runs of homozygosity
*F*_HOM_: Inbreeding coefficient based on homozygosity excess
*F*_GRM_: Inbreeding coefficient based on genomic relationship matrix
*F*_UNI_: Inbreeding coefficient based on uniting gametes
GO: Gene Ontology
Ho: Observed heterozygosity
He: Expected heterozygosity
KEGG: Kyoto Encyclopedia of Genes and Genomes
LD: Linkage disequilibrium
PCA: Principal component analysis
QTL: Quantitative trait loci
*r*: Spearman correlation
REH: Relative humidity
ROH: Runs of homozygosity
SNPs: Single-nucleotide polymorphisms
WGS: Whole-genome sequencing
