# Supplementary material for: Runs of homozygosity reveal population dynamics and selection across global cattle
Source: J Anim Sci Biotechnol. 2026 May 6;17:84. doi: 10.1186/s40104-026-01403-0 (PMC13147640; doi:10.1186/s40104-026-01403-0)
Supplement: Supplementary file 2 — Additional file 2: Fig. S1 Classification of ROH into three size categories using a three-component Gaussian mixture model. Fig. S2 Sensitivity analysis of ROH identification parameters across seven representative cattle breeds with diverse genetic backgrounds. Fig. S3 Landscape of individual ROH cumulative number across four categories in 102 cattle populations. Fig. S4 Principal component analysis based on whole-genome SNP variations. Fig. S5 Admixture analysis for K = 2–20 based on whole-genome SNP variations. Fig. S6 Principal component analysis diagram based on the existence of ROH. Fig. S7 PCA plot based on SNP genotypes within different categories of ROH segments. Fig. S8 Admixture plot for short ROH with K = 2–20. Fig. S9 Admixture plot for long ROH with K = 2–20. Fig. S10 Admixture plot for medium ROH with K = 2–20. Fig. S11 Admixture plot for total ROH with K = 2–20. Fig. S12 Spearman correlation between ROH length and number across four length categories. Fig. S13 Spearman correlation analysis between FROH and observed (Ho) or expected (He) heterozygosity. Fig. S14 UpsetR map of ROH hotspots in cattle genomes from 17 regions. Fig. S15 Manhattan plot of the frequency of SNPs within runs of homozygosity (ROH) across 17 global cattle populations. The dashed line represents the permutation test threshold P < 0.01 used to identify ROH hotspots. Fig. S16 Global allele frequency distribution of NCAPG rs110251642 associated with growth across 102 cattle breeds. Fig. S17 Global allele frequency distribution of CHEK2 rs520226567 associated with cold adaptation across 102 cattle breeds. Fig. S18 Climatic niche distribution of cattle breeds based on mean temperature of the warmest quarter (Bio10) and relative humidity (REH). Fig. S19 Environmental correlation analysis of missense variants within selection-validated heat tolerance ROH hotspots. Fig. S20 Paired Wilcoxon signed-rank test for frequency of ROH located within wild bovine-introgressed (banteng or gaur) regi [file 40104_2026_1403_MOESM2_ESM.pdf]

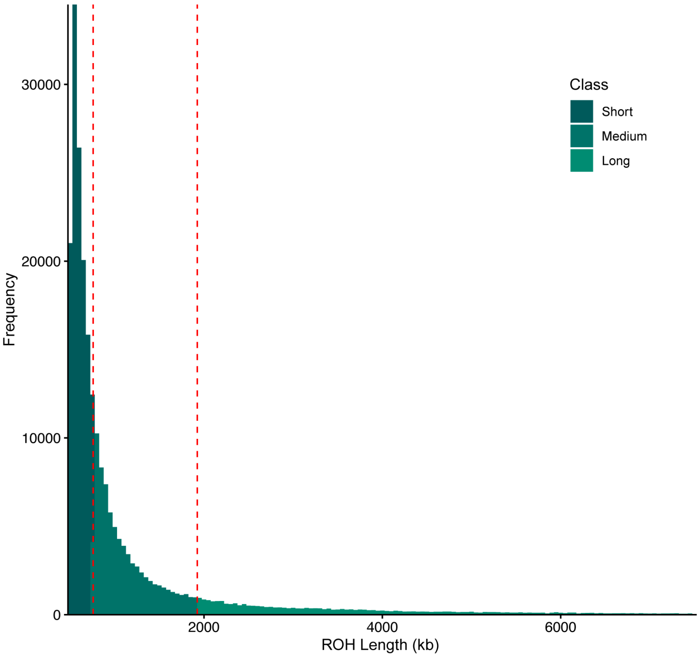

Fig. S1 Classification of ROH into three size categories using a three-component Gaussian mixture model.

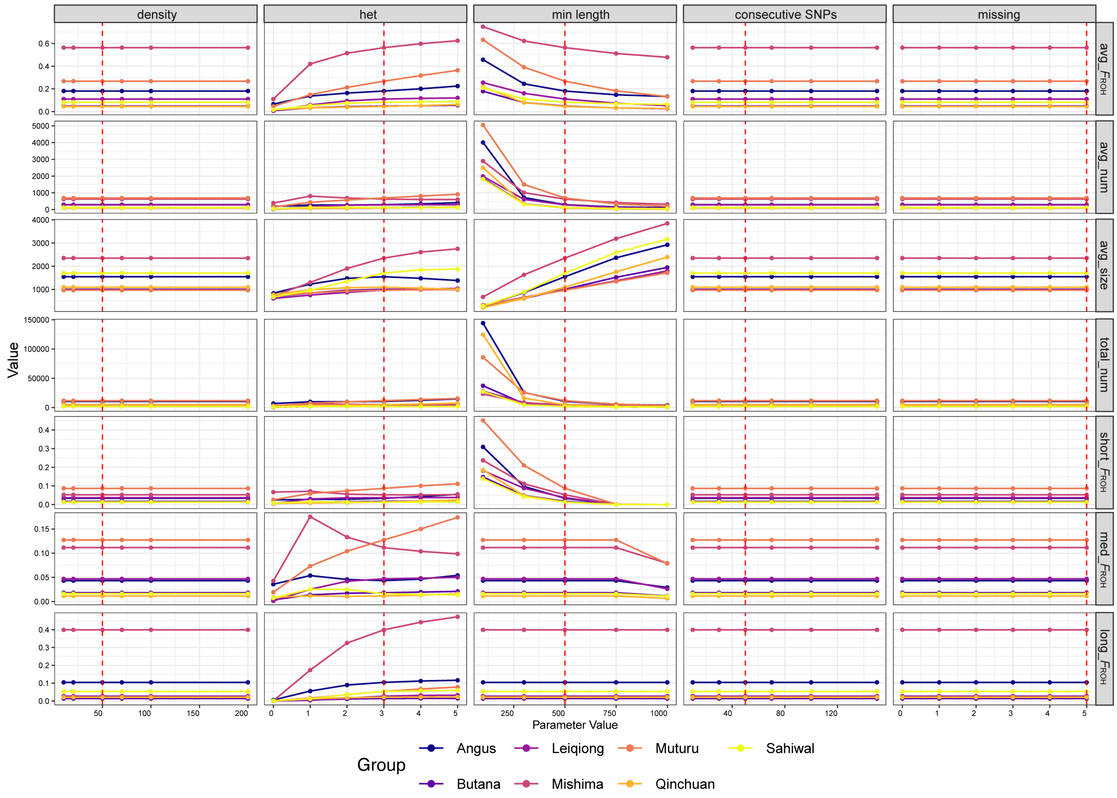

Fig. S2 Sensitivity analysis of ROH identification parameters across seven representative cattle breeds with diverse genetic backgrounds.

The impact of varying ROH detection parameters on key metrics, including SNP density (density), allowed heterozygous genotypes (het), minimum ROH length (min

length, kb), minimum number of consecutive SNPs (consecutive SNPs), and allowed missing genotypes (missing), on various genomic autozygosity metrics. The evaluated metrics (y-axis) include the average ROH-based inbreeding coefficient (avg_F_{ROH}), average number of ROH per individual (avg_num), average ROH size (avg_size, kb), total number of ROH identified (total_num), and the F_{ROH} proportions contributed by short, medium, and long categories (short_F_{ROH}, med_F_{ROH}, and long_F_{ROH}). The red dashed vertical lines indicate the base scenario parameters (snp = 50, minkb = 500, het = 3, miss = 5, density = 50) utilized for the primary analysis in this study.

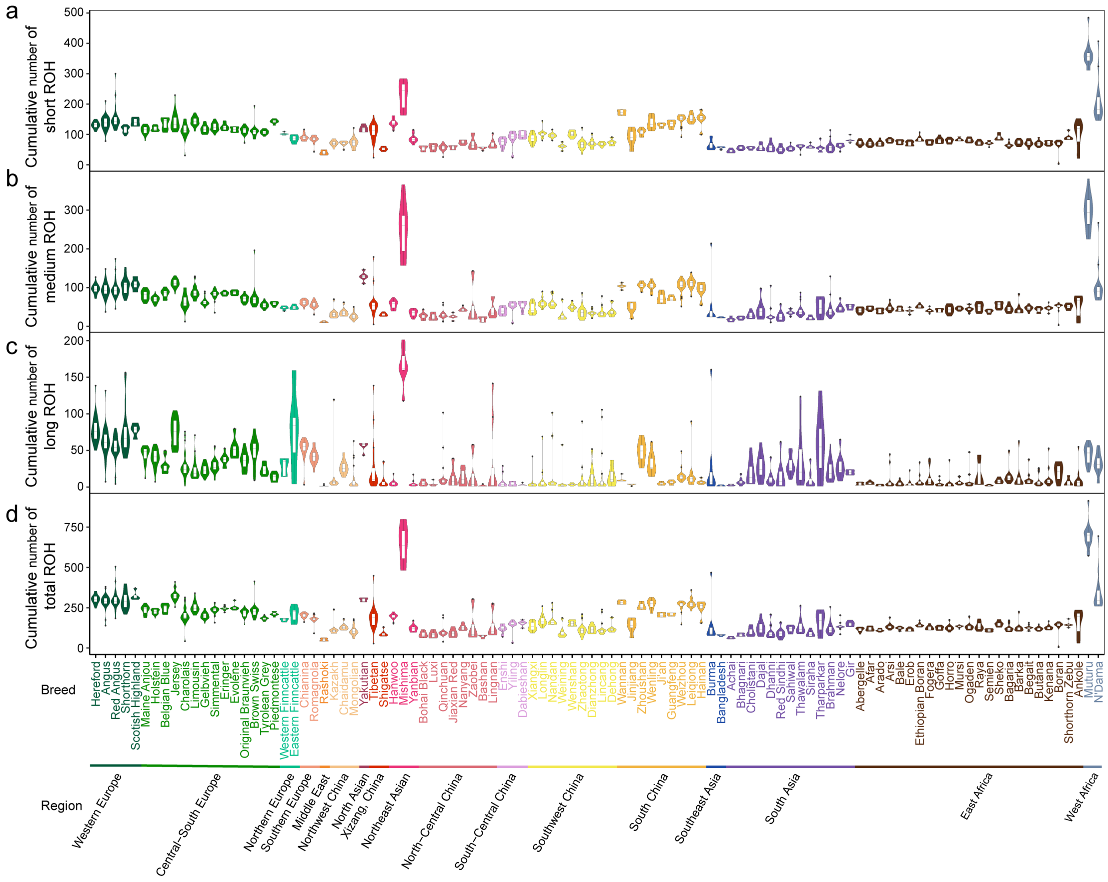

Fig. S3 Landscape of individual ROH cumulative number across four categories in 102 cattle populations.

a short ROH class, **b** medium ROH class, **c** long ROH class, **d** total ROH class.

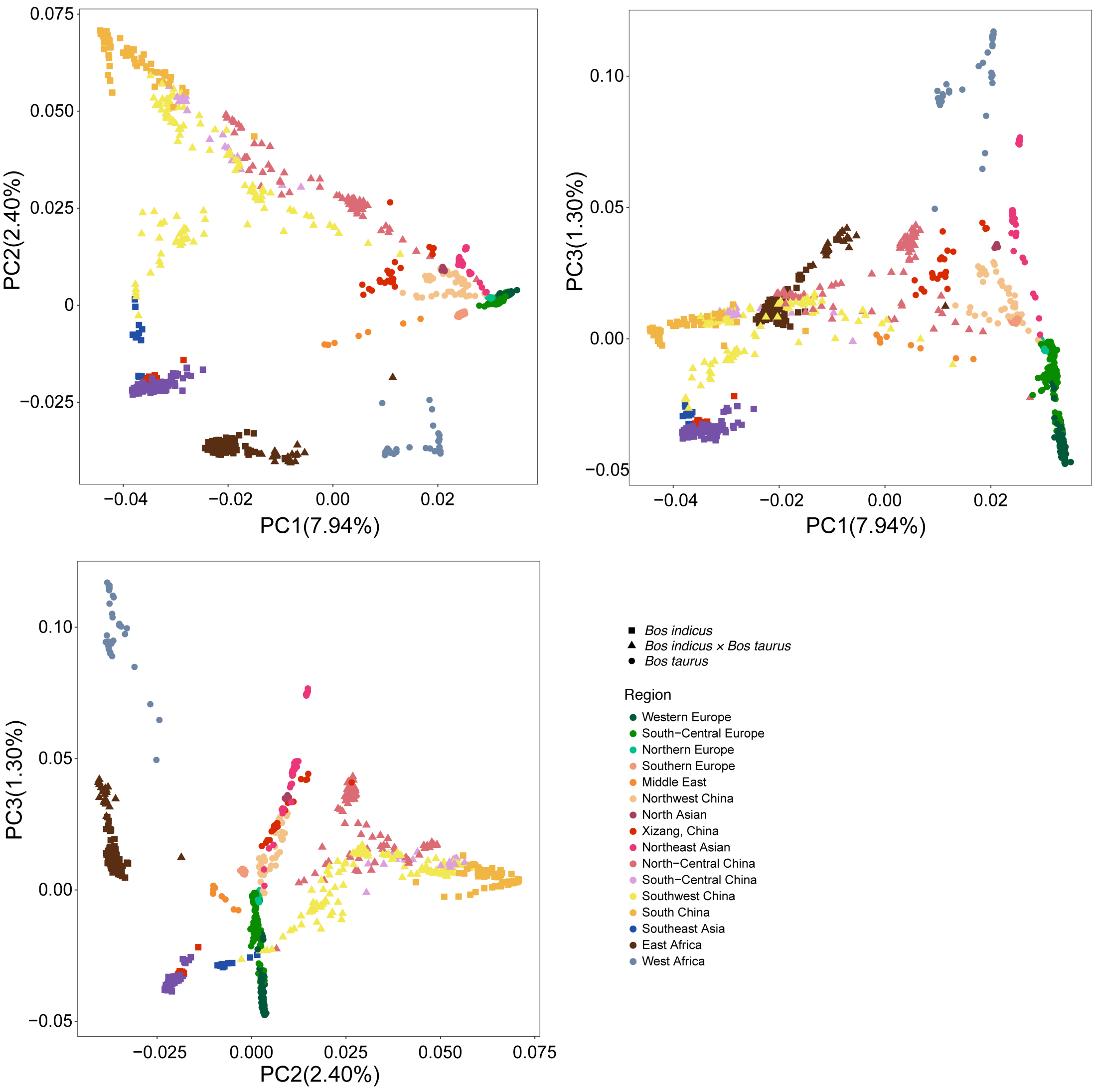

Fig. S4 Principal component analysis based on whole-genome SNP variations.

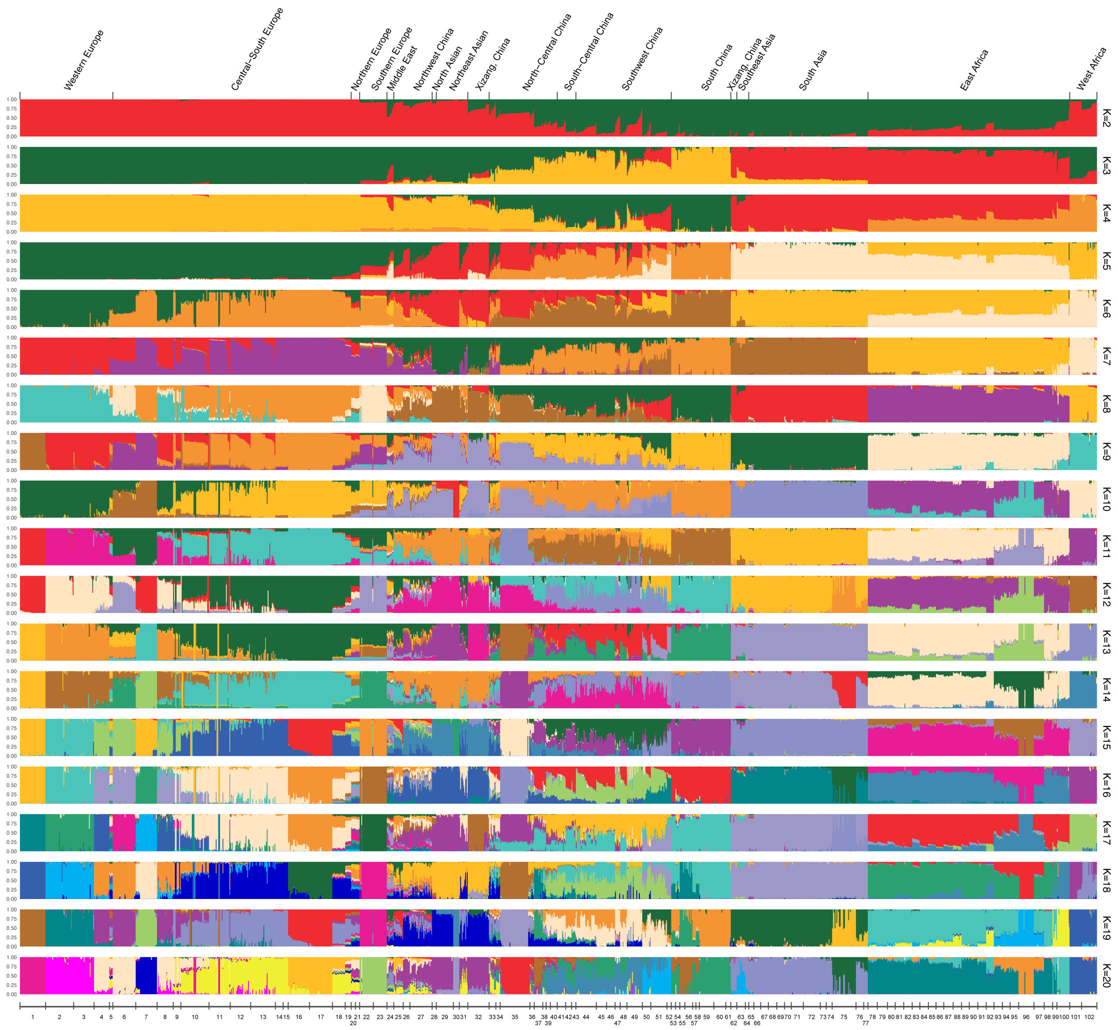

Fig. S5 Admixture analysis for K = 2-20 based on whole-genome SNP variations. The numbers listed in the figure represent (1) Hereford, (2) Angus, (3) Red Angus, (4) Shorthorn, (5) Scotish Highland, (6) Holstein, (7) Jersey, (8) Maine Anjou, (9) Belgian Blue, (10) Charolais, (11) Limousin, (12) Gelbvieh, (13) Simmental, (14) Eringer, (15) Evolène, (16) Original Braunvieh, (17) Brown Swiss, (18) Tyrolean Grey, (19) Piedmontese, (20) Western Finncattle, (21) Eastern Finncattle, (22) Chianina, (23) Romagnola, (24) Rashoki, (25) Kazakh, (26) Chaidamu, (27) Mongolian, (28) Yakutian, (29) Hanwoo, (30) Mishima, (31) Yanbian, (32) Tibetan, (33) Bohai Black, (34) Luxi, (35) Qinchuan, (36) Jiaxian Red, (37) Nanyang, (38) Zaobei, (39) Bashan, (40) Lingnan, (41) Enshi, (42) Yiling, (43) Dabieshan, (44) Xiangxi, (45) Longlin, (46) Nandan, (47) Weining, (48) Wenshan, (49) Zhaotong, (50) Dianzhong, (51) Lincang, (52) Dehong, (53) Wannan, (54) Jinjiang, (55) Zhoushan, (56) Wenling, (57) Ji'an, (58) Guangfeng, (59) Weizhou, (60) Leiqiong, (61) Hainan,

(62) Shigatse, (63) Burma, (64) Bangladesh, (65) Achai, (66) Bhagnari, (67) Cholistani, (68) Dajal, (69) Dhanni, (70) Red Sindhi, (71) Sahiwal, (72) Thawalam, (73) Siraha, (74) Tharparkar, (75) Brahman, (76) Nelore, (77) Gir, (78) Abergelle, (79) Afar, (80) Arado, (81) Arsi, (82) Bale, (83) Erob, (84) Ethiopian Boran, (85) Fogera, (86) Goffa, (87) Horro, (88) Mursi, (89) Ogaden, (90) Raya , (91) Semien, (92) Sheko, (93) Bagaria, (94) Barka, (95) Begait , (96) Butana, (97) Kenana, (98) Boran, (99) Shorthorn Zebu, (100) Ankole, (101) Muturu, (102) N'Dama.

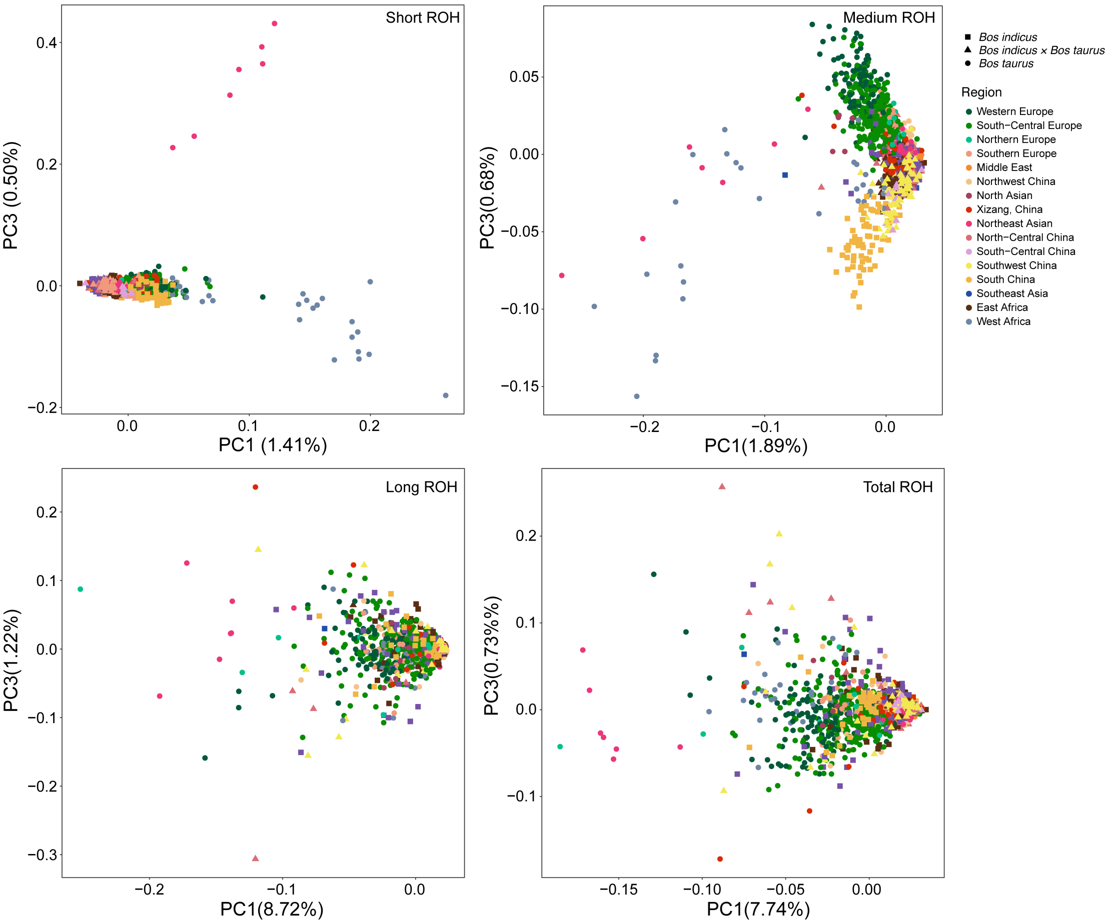

Fig. S6 Principal component analysis diagram based on the existence of ROH. **a** short ROH class, **b** medium ROH class, **c** long ROH class, **d** total ROH class.

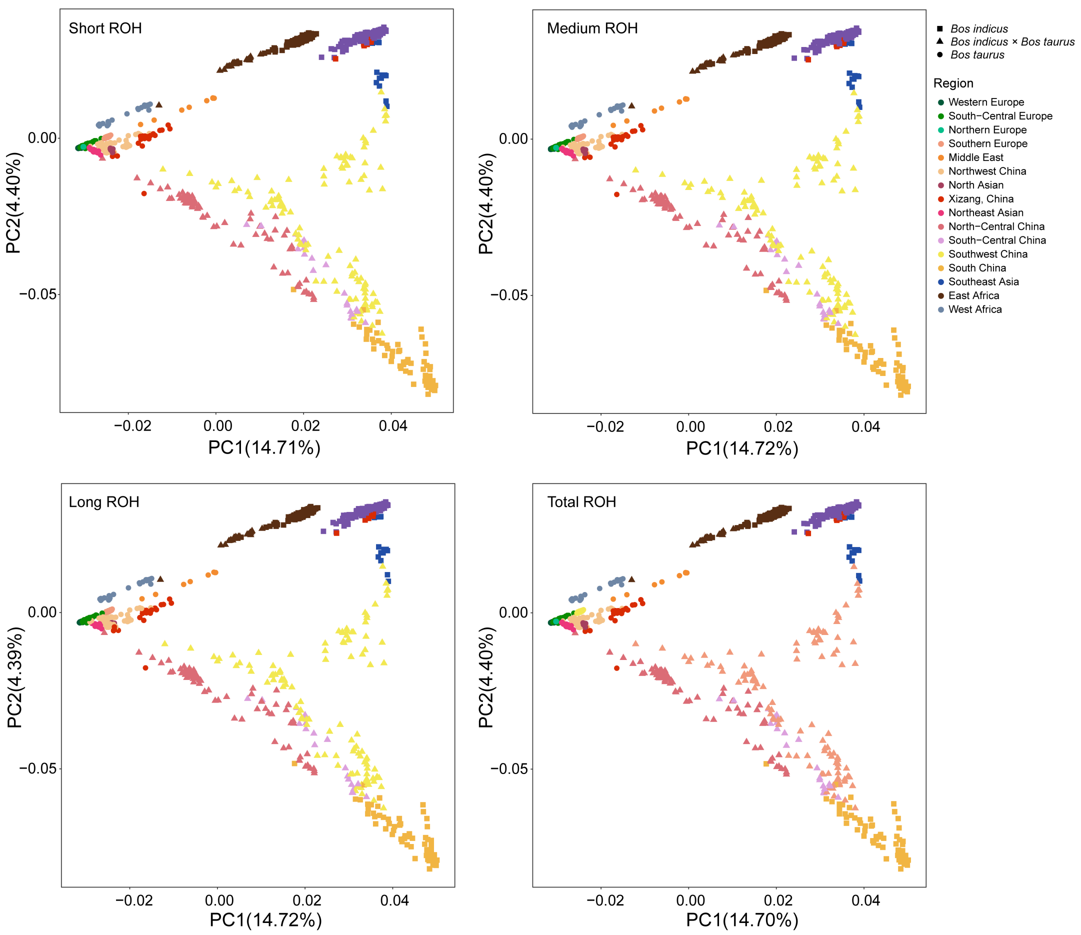

Fig. S7 PCA plot based on SNP genotypes within different categories of ROH segments.

a short ROH class, **b** medium ROH class, **c** long ROH class, **d** total ROH class.

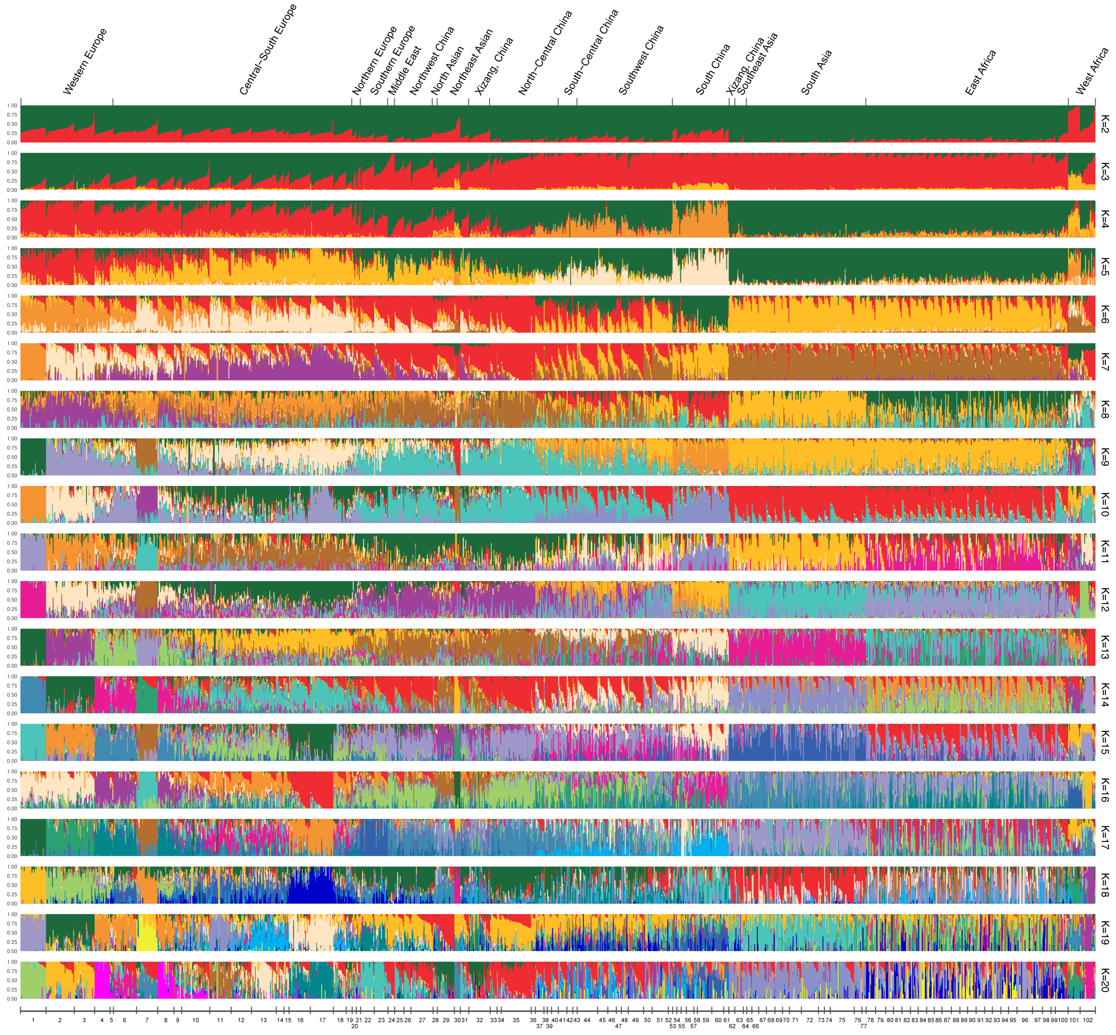

Fig. S8 Admixture plot for short ROH with K = 2-20. Breed numbers on the x-axis correspond to those listed in Fig. S5.

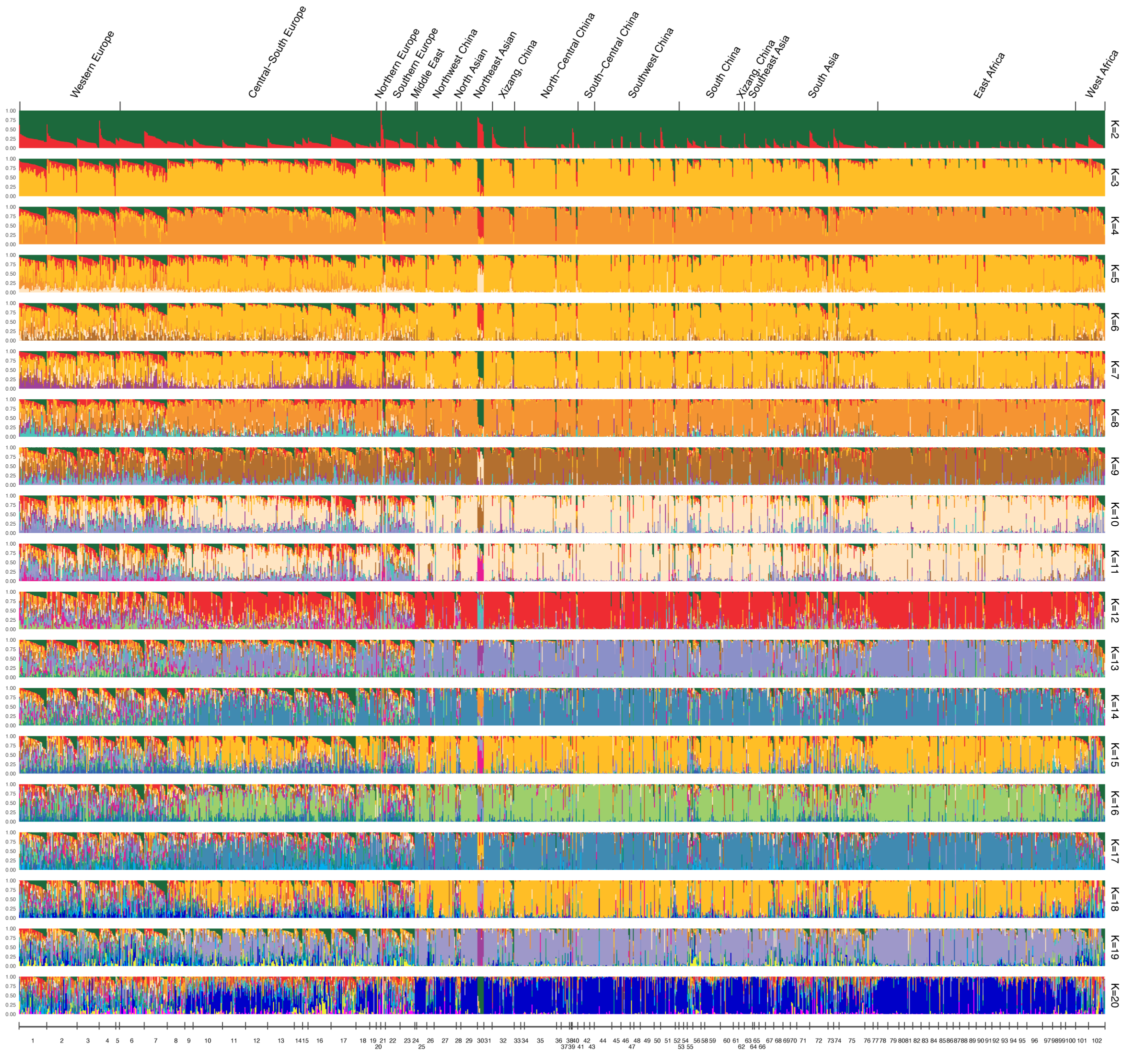

Fig. S9 Admixture plot for long ROH with K = 2-20. Breed numbers on the x-axis correspond to those listed in Fig. S5.

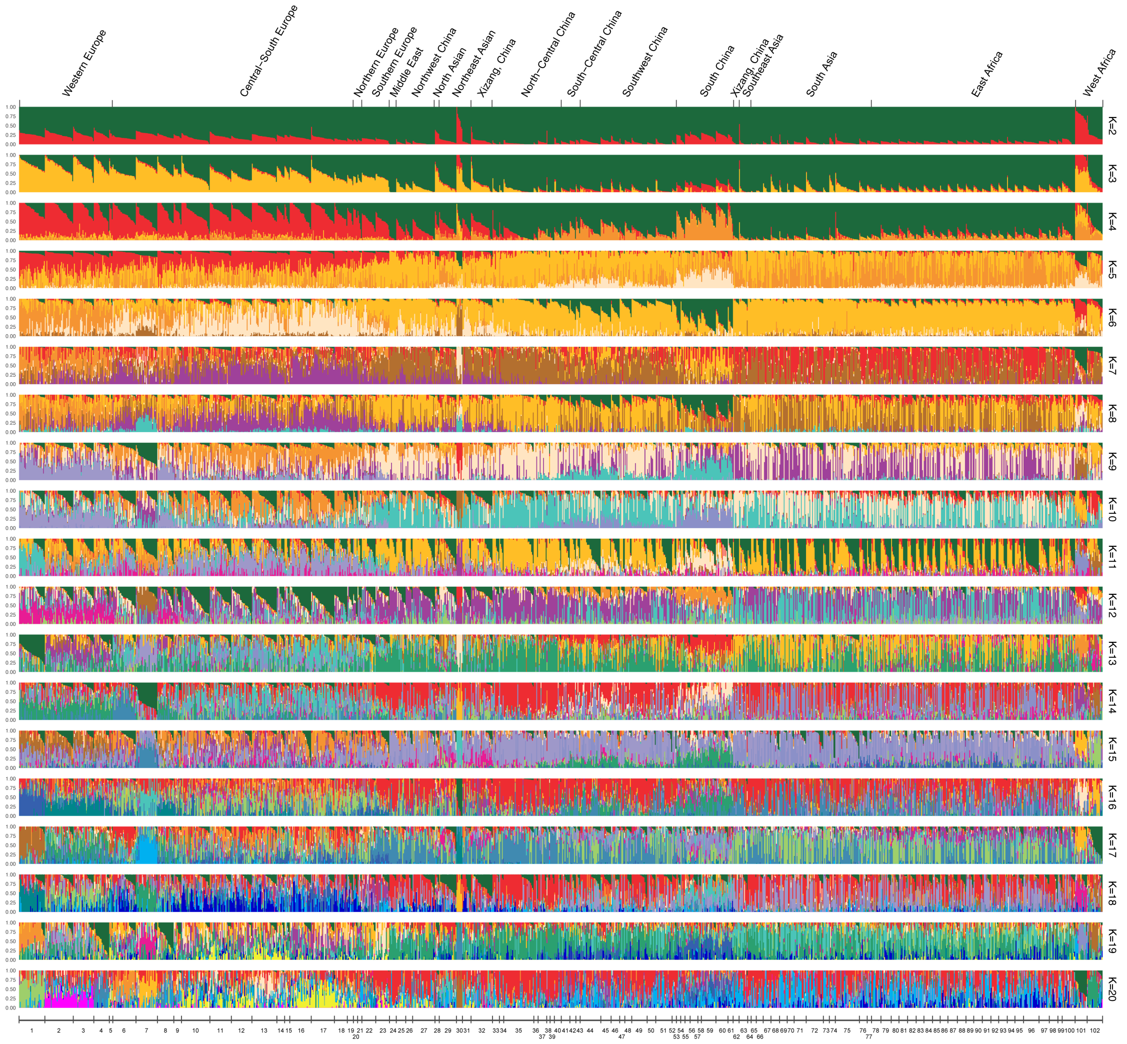

Fig. S10 Admixture plot for medium ROH with K = 2-20. Breed numbers on the x-axis correspond to those listed in Fig. S5.

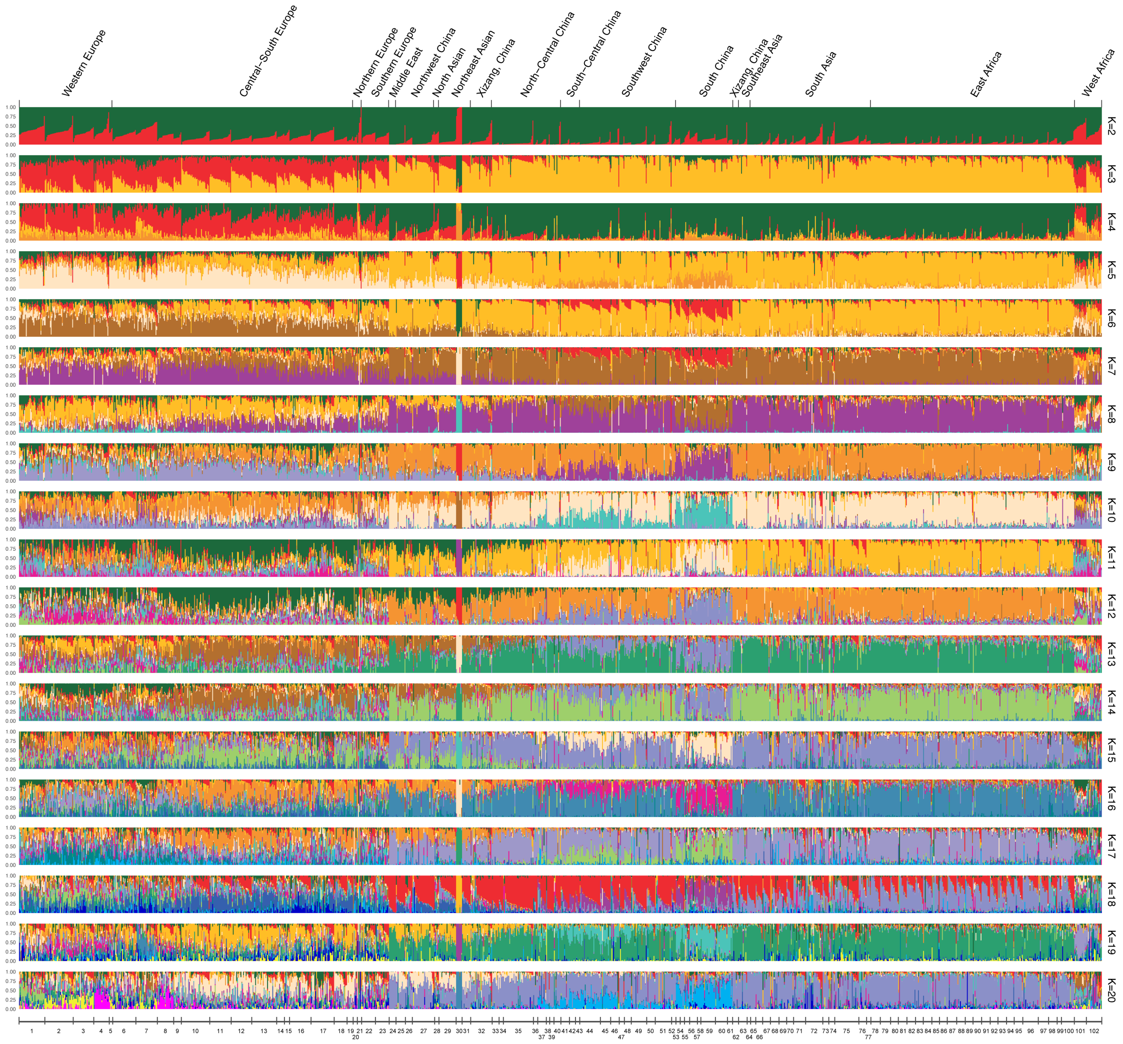

Fig. S11 Admixture plot for total ROH with K = 2-20. Breed numbers on the x-axis correspond to those listed in Fig. S5.

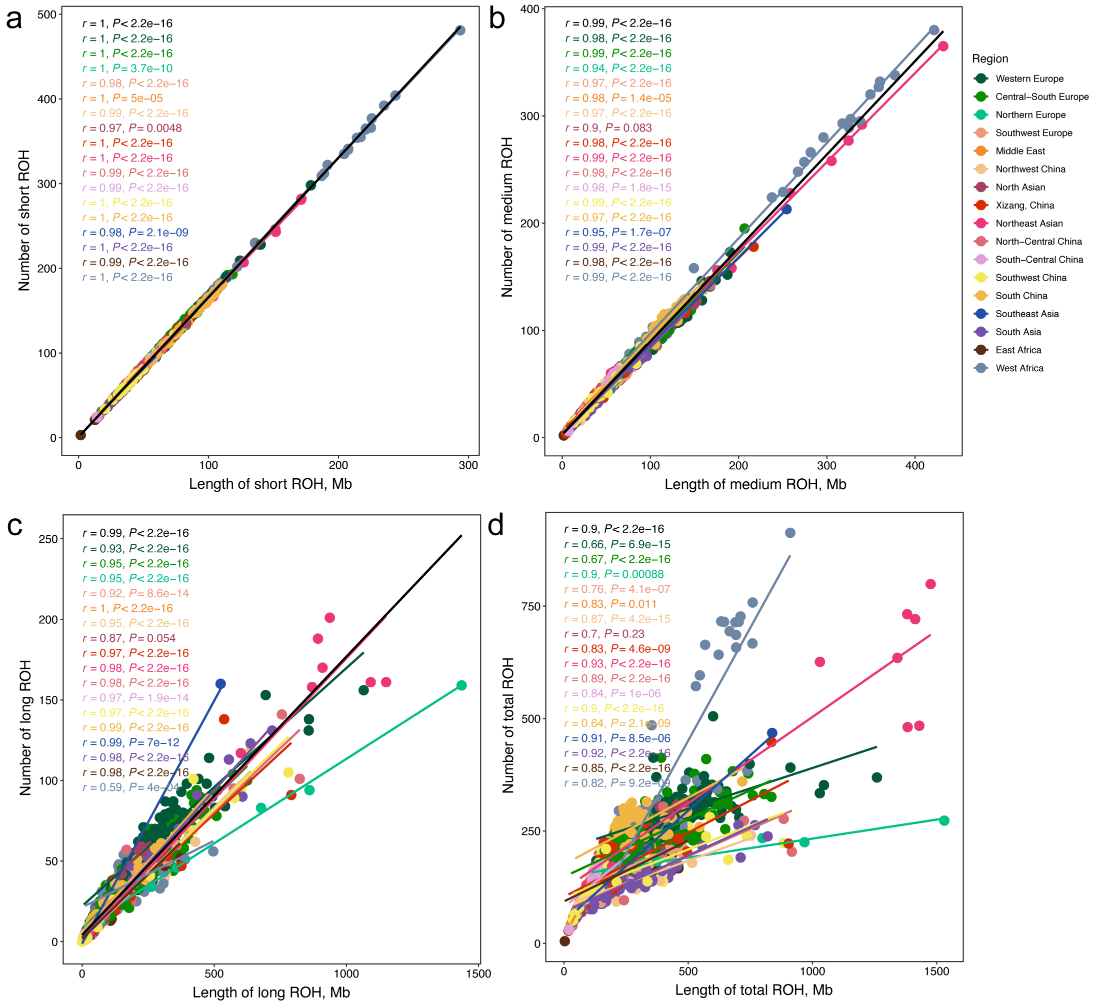

Fig. S12 Spearman correlation between ROH length and number across four length categories.

a Correlation between short ROH length and number, **b.** Correlation between medium ROH length and number, **c.** Correlation between long ROH length and number, **d** Correlation between total ROH length and number.

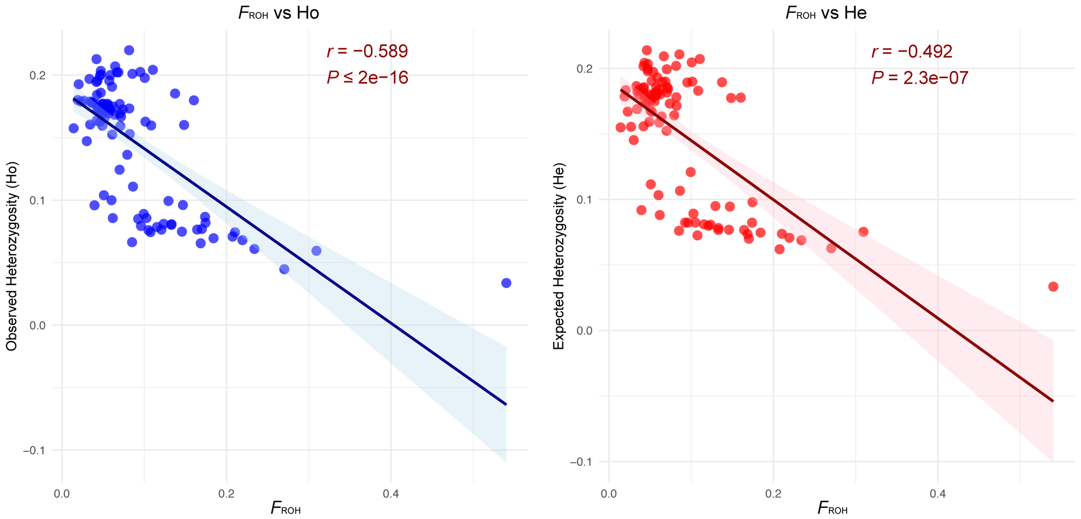

Fig. S13 Spearman correlation analysis between F_{ROH} and observed (Ho) or expected (He) heterozygosity.

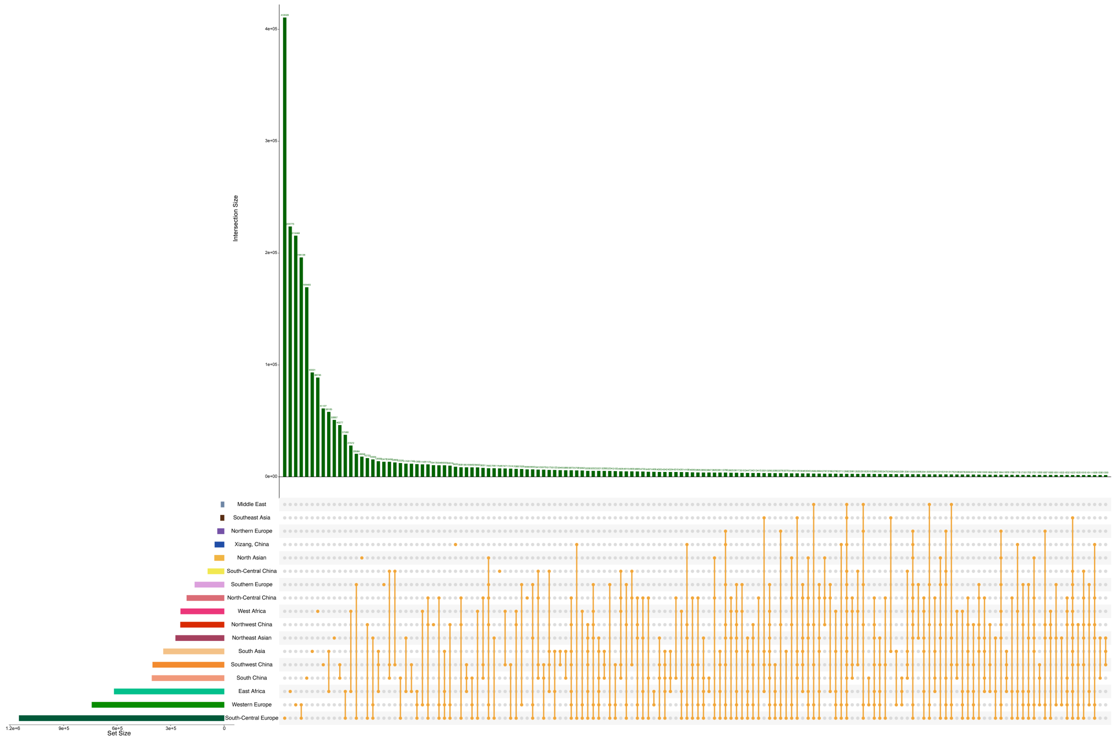

Fig. S14 UpsetR map of ROH hotspot SNPs in cattle genomes from 17 regions.

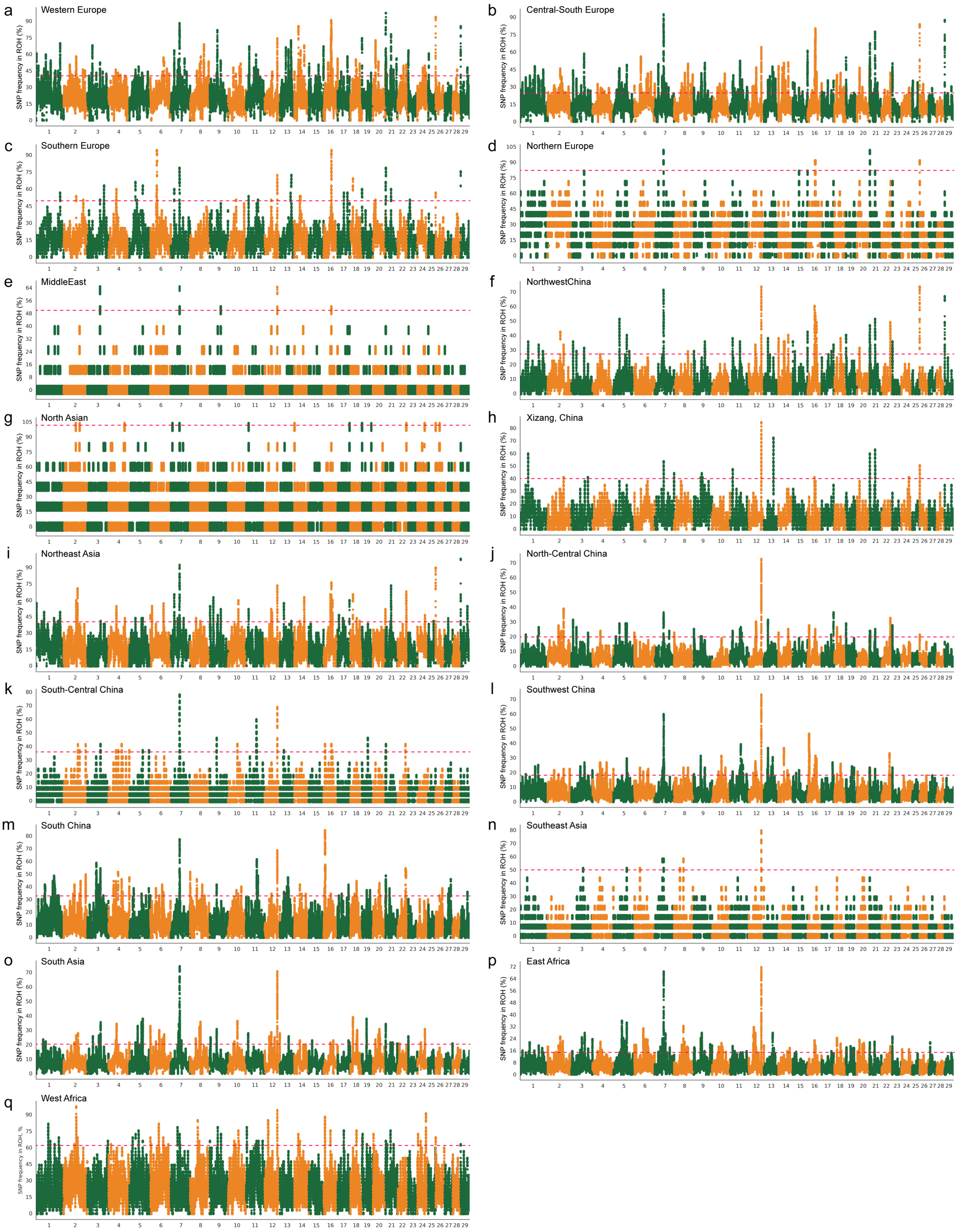

Fig. S15 Manhattan plot of the frequency of SNPs within runs of homozygosity (ROH) across 17 global cattle populations. The dashed line represents the permutation test threshold $P < 0.01$ used to identify ROH hotspots.

a Western Europe. **b** Central-South Europe. **c** Northern Europe. **d** Southern Europe. **e** Middle East. **f** Northwest China. **g** North Asia. **h** Xizang, China. **i** Northeast Asia. **j**

North-Central China. **k** South-Central China. **l** Southwest China. **m** South China. **n** Southeast Asia. **o** South Asia. **p** East Africa. **q** West Africa.

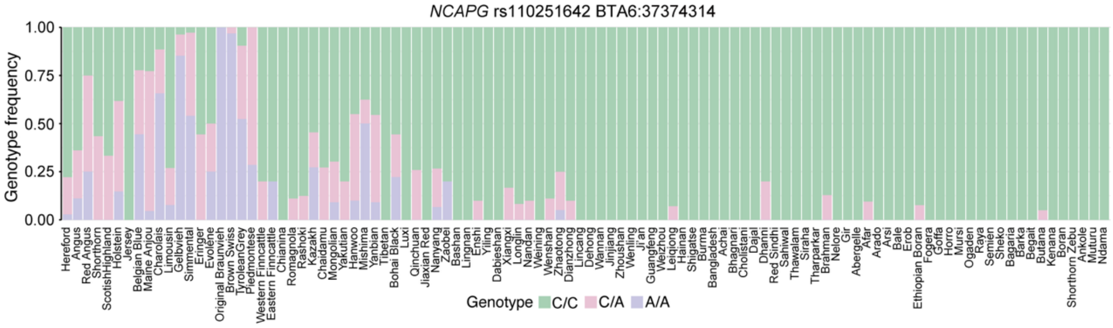

Fig. S16 Global allele frequency distribution of *NCAPG* rs110251642 associated with growth across 102 cattle breeds. In each panel, the stacked bar charts represent the genotype frequencies.

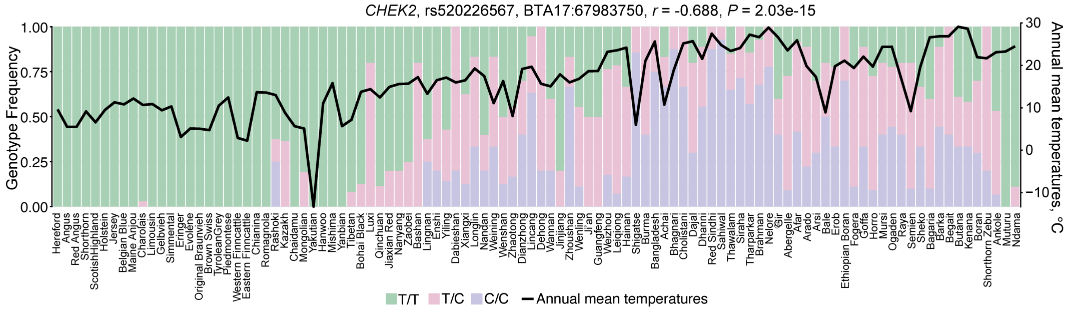

Fig. S17 Global allele frequency distribution of *CHEK2* rs520226567 associated with cold adaptation across 102 cattle breeds. In each panel, the stacked bar charts represent the genotype frequencies (left y-axis). The black lines represent the annual mean temperature (Bio1 value, °C) (right y-axis).

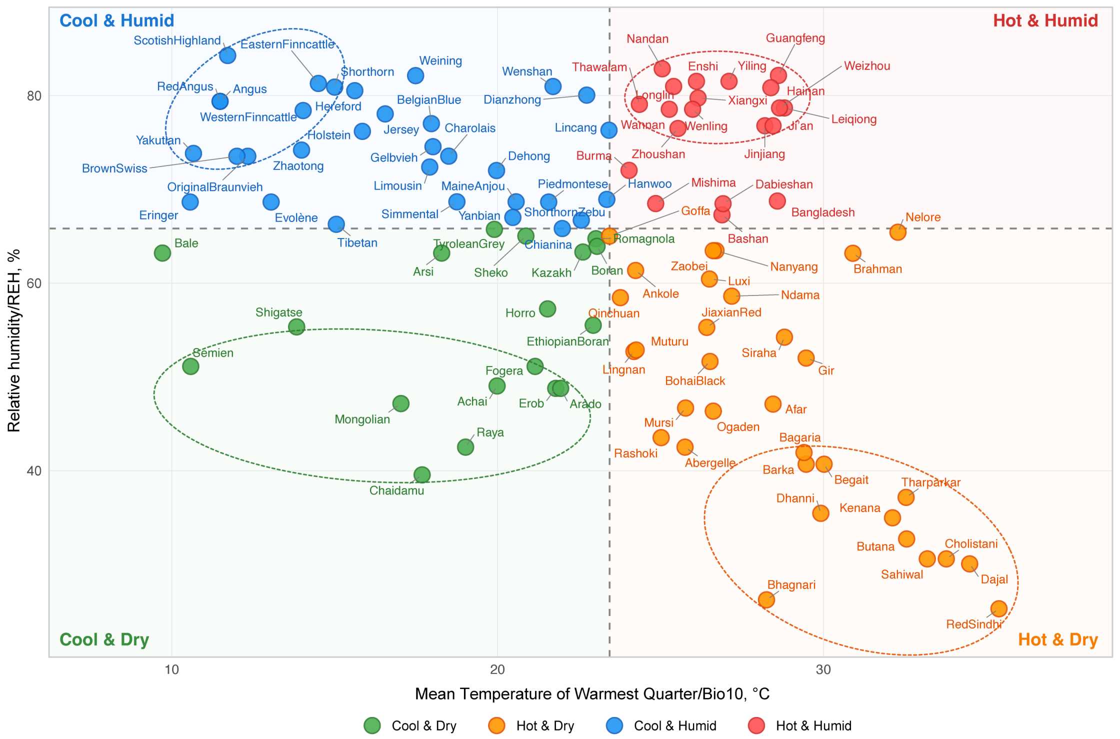

Fig. S18 Climatic niche distribution of cattle breeds based on mean temperature of the warmest quarter (Bio10) and relative humidity (REH). The plot delineates four climatic groups using medians (dashed lines) of mean temperature of the warmest quarter (°C) and relative humidity (%). Each data point represents a breed, with color and position indicating its climatic category: humid-hot (red), arid-hot (orange), humid-cold (blue), and arid-cold (green). The red dashed ellipse encircles breeds selected for humid-hot adaptation analysis, while the orange dashed ellipse highlights those chosen for arid-hot adaptation analysis.

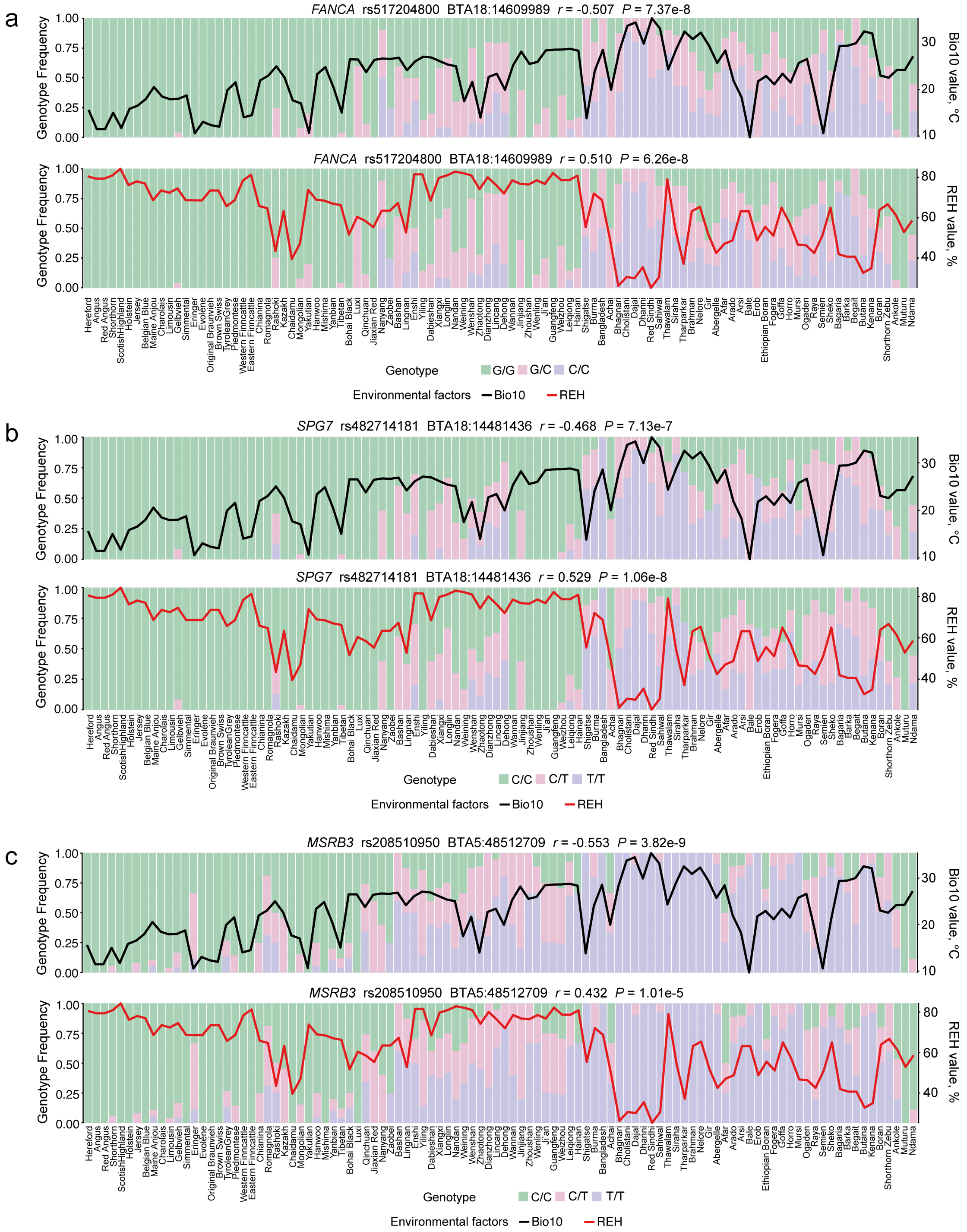

Fig. S19 Environmental correlation analysis of missense variants within selection-validated heat tolerance ROH hotspots.

Distribution of genotype frequencies and their correlation with bioclimatic variables across 102 global cattle breeds for three candidate missense mutations: **a** *FANCA* (rs517204800) on BTA18; **b** *SPG7* (rs482714181) on BTA18; and **c** *MSRB3* (rs208510950) on BTA5. In each panel, the stacked bar charts represent the genotype

frequencies (left y-axis). The black and red lines represent the mean temperature of the warmest quarter (Bio10 value, °C) and the relative humidity (REH value, %), respectively (right y-axis). Spearman's correlation coefficients (*r*) and significance levels (*P*) are indicated at the top of each plot. Cattle breeds are arranged along the x-axis according to their geographic regions and ancestral lineages.

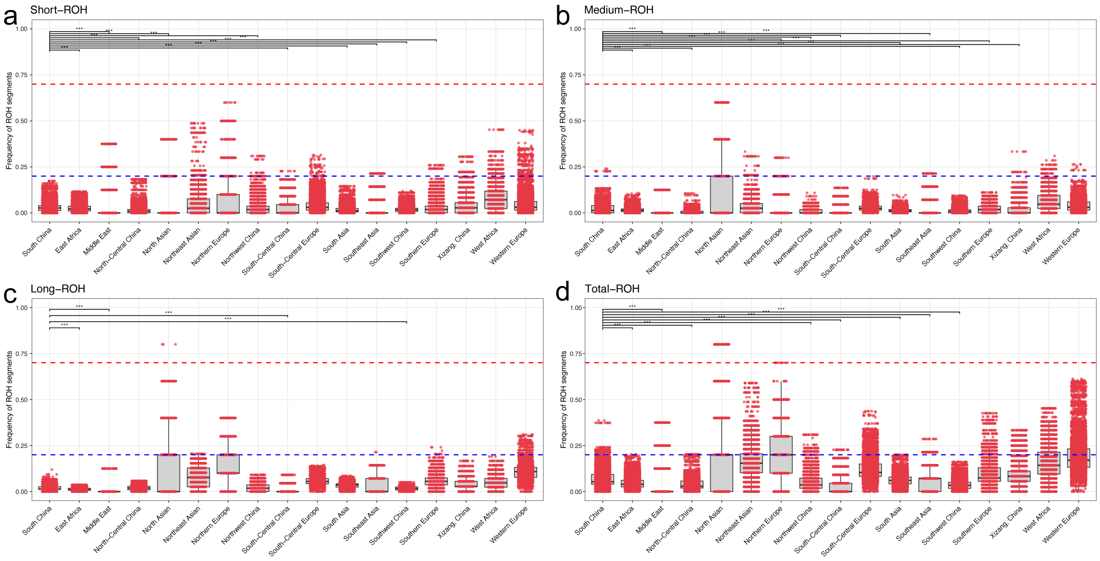

Fig. S20 Paired Wilcoxon signed-rank test for frequency of ROH located within wild bovine-introgressed (banteng and gaur) regions of Southern Chinese indicine cattle from two previous studies.

a short ROH, **b** medium ROH, **c** long ROH, **d** total ROH.

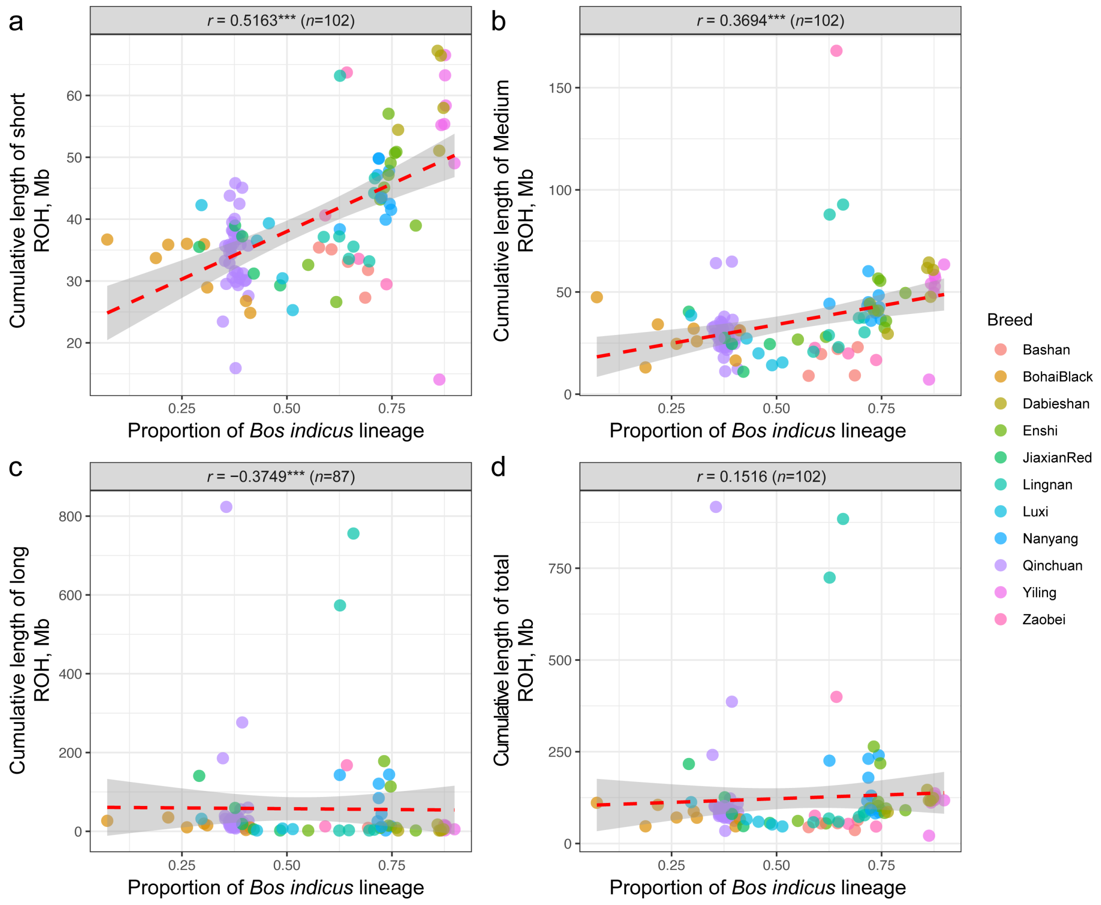

Fig. S21 Scatter plots display Spearman correlations between indicine ancestry proportions (derived from whole-genome SNP Admixture analysis at K = 2) and cumulative lengths of four ROH categories across 125 individuals from 11 cattle breeds in Central China.

a short ROH, **b** medium ROH, **c** long ROH, **d** total ROH. Each point represents an individual, colored by breed. Red dashed lines indicate linear regression fits, with shaded areas representing 95% confidence intervals. Panel titles show Spearman's correlation coefficients (r) and significance levels: $***P < 0.001$, $**P < 0.01$, $*P < 0.05$. The remaining valid samples (n) for each ROH category, excluding zero ROH values, are used for analysis.
